# Psychiatric Disorders Before and After Dementia Diagnosis

**DOI:** 10.1001/jamanetworkopen.2023.38080

**Published:** 2023-10-17

**Authors:** Minjia Mo, Lluis Zacarias-Pons, Minh Tuan Hoang, Shayan Mostafaei, Pol Grau Jurado, Isidora Stark, Kristina Johnell, Maria Eriksdotter, Hong Xu, Sara Garcia-Ptacek

**Affiliations:** 1 Division of Clinical Geriatrics, Department of Neurobiology, Care Sciences and Society, Karolinska Institutet, Stockholm, Sweden; 2 Vascular Health Research Group of Girona (ISV-Girona), Institut Universitari d’Investigació en Atenció Primària Jordi Gol i Gurina (IDIAP Jordi Gol), Girona, Spain; 3 Network for Research on Chronicity, Primary Care, and Health Promotion (RICAPPS), Spain; 4 Department of Medical Epidemiology and Biostatistics, Karolinska Institutet, Stockholm, Sweden; 5 Theme Inflammation and Aging, Karolinska University Hospital, Stockholm, Sweden

## Abstract

**Importance:**

Little is known about the specific timing and sequence of incident psychiatric comorbidities at different stages of dementia diagnosis.

**Objectives:**

To examine the temporal risk patterns of psychiatric disorders, including depression, anxiety, stress-related disorders, substance use disorders, sleep disorders, somatoform/conversion disorders, and psychotic disorders, among patients with dementia before, at the time of, and after receipt of a diagnosis.

**Design, Setting, and Participants:**

This population-based, nationwide cohort study analyzed data from 796 505 participants obtained from 6 registers between January 1, 2000, and December 31, 2017, including the Swedish registry for cognitive/dementia disorders. Patients with dementia were matched on year of birth (±3 years), sex, and region of residence with up to 4 controls. Data were analyzed between March 1, 2023, and August 31, 2023.

**Exposures:**

Any cause of dementia and dementia subtypes.

**Main Outcomes and Measures:**

Flexible parametric survival models to determine the time-dependent risk of initial diagnosis of psychiatric disorders, from 7 years prior to dementia diagnosis to 10 years after diagnosis. Subgroup analysis was conducted for psychiatric drug use among persons receiving a diagnosis of dementia from January 1, 2011, to December 31, 2012.

**Results:**

Of 796 505 patients included in the study (mean [SD] age at diagnosis, 80.2 [8.3] years; 448 869 (56.4%) female), 209 245 had dementia, whereas 587 260 did not, across 7 824 616 person-years. The relative risk of psychiatric disorders was consistently higher among patients with dementia compared with control participants and began to increase from 3 years before diagnosis (hazard ratio, [HR], 1.72; 95% CI, 1.67-1.76), peaked during the week after diagnosis (HR, 4.74; 95% CI, 4.21-5.34), and decreased rapidly thereafter. Decreased risk relative to controls was observed from 5 years after diagnosis (HR, 0.93; 95% CI, 0.87-0.98). The results were similar for Alzheimer disease, mixed dementia, vascular dementia and unspecified dementia. Among patients with dementia, markedly elevated use of psychiatric medications was observed in the year leading up to the dementia diagnosis and peaked 6 months after diagnosis. For example, antidepressant use was persistently higher among patients with dementia compared with controls, and the difference increased from 2 years before dementia diagnosis (15.9% vs 7.9%, *P* < .001), peaked approximately 6 months after dementia diagnosis (29.1% vs 9.7%, *P* < .001), and then decreased slowly from 3 years after diagnosis but remained higher than controls 5 years after diagnosis (16.4% vs 6.9%, *P* < .001).

**Conclusions and Relevance:**

The findings of this cohort study that patients with dementia had markedly increased risks of psychiatric disorders both before and after dementia diagnosis highlight the significance of incorporating psychiatric preventative and management interventions for individuals with dementia across various diagnostic stages.

## Key Points

**Question:**

What are the temporal risk patterns of psychiatric disorders, including depression, anxiety, stress-related disorders, substance use disorders, sleep disorders, somatoform/conversion disorders, and psychotic disorders, before and after dementia diagnosis compared with individuals without dementia?

**Findings:**

In this nationwide cohort study of 796 505 participants conducted in Sweden, compared with participants without dementia, the overall risk of new onset psychiatric disorders was significantly higher among patients with dementia. The risk increased markedly from 3 years before receipt of dementia diagnosis, peaked during the week after diagnosis, and then declined rapidly.

**Meaning:**

These results suggest that managing psychiatric comorbidities is crucial for individuals with dementia across various disease stages.

## Teaser

This nationwide cohort study conducted in Sweden compares the temporal risk patterns of psychiatric disorders before, at the time of, and after a dementia diagnosis between patients without vs with dementia.

## Introduction

Dementia was estimated in 2016 to cause 28.8 million disability-adjusted life years across all ages^1^ and is the second largest cause of death among individuals older than 70 years.^2^ Increased longevity has led to an increasing number of individuals living with dementia and a significant burden on social welfare, health care, and financial systems.^3^ Persons with dementia have elevated rates of premature mortality^4^ and increased risk of suicide,^5^ especially among patients with dementia having psychiatric comorbidities.^6,7^

The co-occurrence of dementia and psychiatric disorders, such as depression and anxiety, are common in clinical practice.^8,9^ While there is substantial research on depression,^10,11^ most of the evidence is limited to the period prior to dementia diagnosis.^12^ There has been comparatively little investigation addressing the period immediately following a dementia diagnosis. Furthermore, other psychiatric disorders, including substance use disorders,^9^ stress reaction or adjustment disorders,^13^ and sleep disorders,^14^ have received less attention.

Accumulating evidence suggests that the period during the receipt of a diagnosis of dementia can be a difficult.^15^ However, evidence regarding how psychiatric disorders develop after a dementia diagnosis is limited by small sample sizes and short follow-ups.^16,17^ A longitudinal description characterizing the burden of psychiatric disorders among individuals with dementia is lacking. Moreover, previous findings relating to rarer dementias, such as frontotemporal dementia (FTD),^18^ dementia with Lewy bodies,^14,19,20^ and Parkinson disease dementia^21^ have been inconclusive due to small sample size, cross-sectional study design, and a short follow-up period. The time-dependent risk patterns of incident psychiatric disorders at different stages of dementia are largely unknown. Information on such conditions may improve timely assessment and early intervention in this growing group of patients.

In this cohort study, we examined the hypothesis that patients with dementia are at increased risk of psychiatric disorders both before and after dementia diagnosis.

## Methods

### Study Design

Based on nationwide Swedish registers, we conducted a population-based cohort study between January 1, 2000, and December 31, 2017, to compare the time-dependent risk of psychiatric disorders between patients with dementia and individuals without dementia during the periods before and after receipt of dementia diagnosis. The requirement for written informed consent for this study was waived due to the register data being pseudonymized before delivery to our research group. The regional ethics committee in Stockholm approved the study, which complied with the Declaration of Helsinki.^22^ Participants and caretakers were informed verbally and in writing about the Swedish Registry for Cognitive/Dementia Disorders (SveDem) and could decline participation. This study adhered to the Strengthening the Reporting of Observational Studies in Epidemiology (STROBE) reporting guideline.

#### Patients With Dementia

Using SveDem, the National Patient Register, and the Swedish Prescribed Drug Register, we identified 223 726 patients (≥40 years old) with a first diagnosis of dementia between May 1, 2007, and December 31, 2017. Details of the 3 register database are presented in the eMethods in Supplement 1.

Because SveDem contains records since 2007 and the National Patient Register contains records on both inpatient and outpatient care since 2001, the maximum time for obtaining psychiatric comorbidities before dementia diagnosis for patients diagnosed in 2007 was 7 years. For this reason, we defined the prediagnostic period as 7 years before the date of dementia diagnosis. The postdiagnostic period was defined as the period from the date of dementia diagnosis onward. To examine the association of dementia disorder progression with the development of new psychiatric disorders, we excluded 14 481 patients who had pre-existing psychiatric comorbidities at the time of enrollment in the cohort (ie, 7 years before the date of diagnosis), leaving 209 245 patients with dementia included in this study. We defined the index date as the date of first diagnostic record of a dementia diagnosis for patients with dementia.

#### Control Participants

The control participants were selected from the Total Population Register. For each individual with dementia, up to 4 control individuals were selected based on year of birth (±3 years), sex, and region of residence. Control participants were excluded if they were not free of psychiatric comorbidities at entry to the cohort (ie, 7 years before the date of the dementia diagnosis receipt of the matched individuals with dementia). For control participants, the same index date as the matched individual with dementia was assigned.

The study followed up all participants from 7 years prior to the index date (earliest possible entry on January 1, 2000), until a first diagnosis of psychiatric disorder, death, or December 31, 2017, whichever occurred first.

### Dementia Diagnosis

Inclusion criteria were as follows: diagnosis of dementia in SveDem; or first record of a dementia defined using the *International Statistical Classification of Diseases and Related Health Problems, Tenth Revision *(*ICD-10*) codes F00 to F03, G30, and G31 in the National Patient Register; or record of the Anatomical Therapeutic Chemical classification code N06D (dementia medication) in the Prescribed Drug Register of Sweden (eTable 1 in Supplement 1). For patients with dementia identified from SveDem, dementia diagnoses were coded as Alzheimer disease (AD), mixed dementia, vascular dementia, dementia with Lewy bodies, FTD, Parkinson disease dementia, and unspecified dementia. Dementia with Lewy bodies and Parkinson disease dementia were merged for this study as Lewy body disease (LBD) considering the shared pathological and clinical characteristics.^23,24^

### Psychiatric Disorders and Medication

We used *ICD-10* codes from the National Patient Register to identify any first inpatient or outpatient diagnosis of common psychiatric disorders during follow-up. Psychiatric disorders included depression, anxiety, stress-related disorders, substance use disorders, sleep disorders, somatoform/conversion disorders, and psychotic disorders (eTable 2 in Supplement 1). To capture conditions that may not be documented in the National Patient Register, we performed a subgroup analysis to examine the use of psychiatric medications associated with psychiatric disorders as assessed by dispensation of prescription medication (eMethods and eTable 3 in Supplement 1).

### Covariates

Socioeconomic characteristics, including educational attainment, marital status, disposable individual income, region of birth, and coresident status were extracted from the longitudinal integrated database for health insurance and labor market studies. The dates of death were extracted from the Cause of Death Register.

Common and major physical disorders diagnosed within 3 years before the index date were identified by *ICD-10* codes from the National Patient Register. We used the Charlson Comorbidity Index (CCI) score^25^ (0, 1, 2, and ≥3, with higher values indicating more comorbidities) to assess medical comorbidities, using a weighted sum of diagnosed chronic disorders^26^ but excluding dementia.

### Statistical Analysis

We first compared the characteristics of patients with dementia and the control participants at the index date. We then investigated the time-dependent associations between dementia diagnosis and the risk of psychiatric disorders by flexible parametric survival models (Royston-Parmar models).^27^ A spline with 5 *df* was used for the baseline rate, while 3 *df* was used for the time-varying effect. We used time since cohort entry as the underlying timescale. Hazard ratios (HRs) and 95% CIs were estimated separately for prediagnostic and postdiagnostic periods. We excluded 26 564 patients with dementia and 34 083 control participants diagnosed as having a psychiatric disorder during the prediagnostic period from the analysis of the postdiagnostic period. The corresponding 72 459 controls of 26 564 patients with dementia were also excluded (eFigure 1 in Supplement 1). Models were adjusted for age, sex, educational attainment, disposable individual income, region of birth, CCI score, and calendar year of dementia. The analysis was applied first to any psychiatric disorders and then for different types of psychiatric disorders. In addition, the HRs for dementia subtypes, including AD, vascular dementia, mixed dementia, FTD, LBD, and unspecified dementia, were examined separately.

In stratified analyses, HRs at each time point were estimated by age (<65, 65-74, and ≥75 years), sex (male or female), educational attainment, CCI score, calendar year of diagnosis, and coresident status (cohabiting or living alone). For patients from SveDem, we also conducted stratified analysis by Mini-Mental State Examination (MMSE) score (0-9, 10-19, 20-24, and 25-30, with higher scores indicating milder cognitive impairment) and type of diagnostic unit (specialist care or primary care). In sensitivity analyses, we repeated the main analyses among patients from SveDem who were considered to have clinically verified diagnoses and patients with presumptive dementia from other registers.

To compare the absolute risk of psychiatric disorders between patients with vs without dementia, we plotted cumulative incidence curves for each individual disorder. Considering a high mortality among patients with dementia after diagnosis, competing risk models were applied in the postdiagnostic period.

The proportions for receiving treatment of the studied psychiatric medications from 5 years before dementia diagnosis to 5 years after dementia diagnosis were calculated daily for patients with dementia and control participants. The differences between the 2 groups were examined by χ^2^ tests with Bonferroni correction for multiple testing. Statistical analyses were conducted from March 1, 2023, to August 31, 2023, with SAS, version 9.4 (SAS Institute Inc) and R 4.2.1 (R Foundation for Statistical Computing), with statistical tests using a 2-tailed *P* < .05 or 95% CI excluding 1 as the level of statistical significance.

## Results

In total, 796 505 patients—209 245 patients with dementia and 587 260 matched controls—were included in this cohort study. The mean (SD) age at the index date was 80.2 (8.3) years; 448 869 patients (56.4%) were female, and 347 636 patients (43.6%) were male (eTable 4 in Supplement 1). Overall, across 7 824 616 person-years, 25 061 individuals with dementia (incidence rate [IR], 17.9 per 1000 person-years) and 34 083 control participants (IR, 8.5 per 1000 person-years) were identified as receiving a new diagnosis of a psychiatric disorder during the prediagnostic period, whereas 20 359 individuals with dementia (IR, 38.5 per 1000 person-years) and 31 034 control participants (IR, 15.3 per 1000 person-years) were identified as receiving a new diagnosis of a psychiatric disorder during the postdiagnostic period.

Compared with that among control participants, the cumulative incidence of most psychiatric disorders (eg, depression IR, 3.4 vs 9.6 per 1000 person-years; anxiety IR, 2.2 vs 4.6 per 1000 person-years), except for sleep disorders (IR, 2.2 vs 2.3 per 1000 person-years) and somatoform/conversion disorders (IR, 0.4 vs 0.5 per 1000 person-years), was higher among patients with dementia, with the highest cumulative incidence observed for depression (eFigure 2 and eTable 5 in Supplement 1). Similar results were noted among patients from different registers (eTable 6 and eTable 7 in Supplement 1). Patients diagnosed as having LBD (IR, 10.2 vs 26.8 per 1000 person-years) and FTD (IR, 11.1 vs 28.7 per 1000 person-years) had higher incidence for overall psychiatric disorders, except sleep disorders, compared with other subtypes (eTable 8 in Supplement 1).

**Figure 1.**
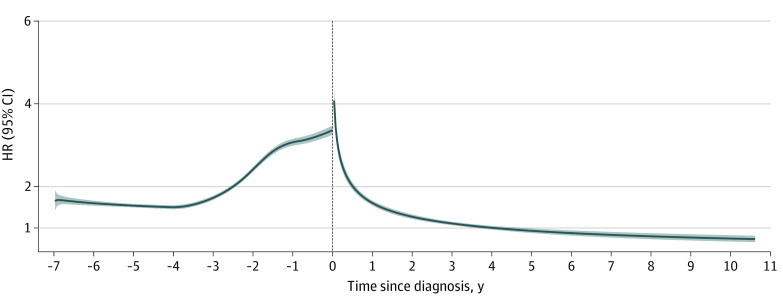
Hazard Ratios (HRs) and 95% CIs of Psychiatric Disorders Before and After Dementia Diagnosis in a Matched Cohort Study in Sweden, 2000 to 2017

The overall relative risk of studied psychiatric disorders was consistently higher in patients with dementia compared with control participants and started to increase from 3 years before dementia diagnosis (HR, 1.72; 95% CI, 1.67-1.76). The HRs reached a peak during the week after diagnosis (4.74; 95% CI, 4.21-5.34). After that, the increase in risk decreased rapidly, and a decreased risk relative to controls was observed from 5 years after diagnosis (HR, 0.93; 95% CI, 0.87-0.98) (Figure 1). Similar results were observed among patients from SveDem and other registers (eFigure 3 and eFigure 4 in Supplement 1).

Compared with patients with dementia who were older, were less educated, and had higher CCI scores, the rate increase of having psychiatric disorders before dementia diagnosis was greater among those who were younger (eg, 0.5 years before diagnosis, ≥75 years of age HR, 3.15 [95% CI, 3.07-3.25]; vs <65 years HR, 6.27 [95% CI, 5.61-7.01]), better educated (eg, 0.5 years before diagnosis, <9 years of education: HR, 2.60 [95% CI, 2.49-2.72] vs ≥13 years of education: HR, 3.90 [95% CI, 3.64-4.19]), and had lower CCI scores (eg, 0.5 years before diagnosis, CCI 0: HR, 4.75 [95% CI, 4.54-4.97] vs ≥3 HR, 1.98 [95% CI, 1.87-2.11]) (Table 1). Similar results were observed for the postdiagnostic period from the index date to 5 years after diagnosis (Table 2). The HRs remained elevated by year 10 after diagnosis among younger patients (HR, 1.51; 95% CI, 1.11-2.07). Furthermore, compared with patients with lower MMSE scores who received a diagnosis in primary care, patients with higher MMSE scores (eg, 0.5 years before diagnosis MMSE score 0-9: HR, 2.14 [95% CI, 1.53-2.98] vs MMSE score 25-30 HR, 3.86 [95% CI, 3.51-4.25]; and 10 years after diagnosis MMSE score 0-9: HR, 0.44 [95% CI, 0.11-1.73] vs MMSE score 25-30 HR, 1.03 [95% CI, 0.78-1.36]) who received a diagnosis in specialist care (eg, 0.5 years before diagnosis in specialist care HR, 4.19 [95% CI, 3.95-4.46] vs diagnosis in primary care HR, 2.24 [95% CI, 2.07-2.44]; and 10 years after diagnosis HR, 0.70 [95% CI, 0.57-0.87] vs diagnosis in primary care HR, 0.73 [95% CI, 0.56-0.95]) had increased risk of diagnosis of new psychiatric disorders both before and after dementia diagnosis (Table 1 and Table 2).

**Table 1.** Hazard Ratios and 95% CIs of Psychiatric Disorders Among Patients With vs Those Without Dementia Before Dementia Diagnosis, by Participant Characteristics^a^

Variable	Risk before diagnosis, HR (95% CI)					
	−6.5 y	−5 y	−3 y	−2 y	−1 y	−0.5 y
Overall	1.63 (1.56-1.70)	1.53 (1.49 to 1.57)	1.72 (1.67 to 1.76)	2.40 (2.35-2.45)	3.05 (2.97-3.13)	3.15 (3.07-3.25)
Age at diagnosis, y						
<65	2.26 (1.95-2.62)	2.11 (1.93-2.30)	2.84 (2.59-3.11)	4.43 (4.08-4.81)	5.84 (5.30-6.44)	6.27 (5.61-7.01)
65-74	2.06 (1.86-2.29)	1.90 (1.79-2.02)	2.25 (2.12-2.40)	3.45 (3.27-3.63)	4.55 (4.28-4.84)	4.75 (4.44-5.09)
≥75	1.44 (1.37-1.52)	1.36 (1.32-1.41)	1.49 (1.45-1.54)	2.03 (1.98-2.08)	2.55 (2.48-2.63)	2.63 (2.55-2.72)
Sex						
Male	1.67 (1.56-1.78)	1.51 (1.45-1.57)	1.71 (1.64-1.78)	2.42 (2.34-2.50)	3.04 (2.93-3.17)	3.14 (3.01-3.28)
Female	1.58 (1.49-1.68)	1.53 (1.48-1.58)	1.70 (1.64-1.76)	2.36 (2.29-2.42)	3.02 (2.92-3.12)	3.13 (3.02-3.26)
Educational attainment, y						
<9	1.54 (1.43-1.66)	1.44 (1.38-1.50)	1.56 (1.49-1.63)	2.07 (2.00-2.14)	2.54 (2.44-2.65)	2.60 (2.49-2.72)
9-12	1.70 (1.59-1.82)	1.59 (1.53-1.65)	1.81 (1.73-1.88)	2.59 (2.51-2.68)	3.35 (3.22-3.49)	3.48 (3.33-3.64)
≥13	1.67 (1.49-1.87)	1.58 (1.48-1.68)	1.90 (1.78-2.03)	2.80 (2.66-2.95)	3.69 (3.47-3.93)	3.90 (3.64-4.19)
Charlson Comorbidity Index^b^						
0	1.90 (1.78-2.03)	1.66 (1.59-1.72)	2.17 (2.09-2.25)	3.65 (3.52-3.78)	4.56 (4.38-4.75)	4.75 (4.54-4.97)
1	1.69 (1.52-1.87)	1.49 (1.41-1.57)	1.46 (1.37-1.55)	1.85 (1.77-1.93)	2.41 (2.28-2.55)	2.50 (2.35-2.66)
2	1.52 (1.34-1.72)	1.48 (1.38-1.58)	1.57 (1.46-1.69)	1.97 (1.87-2.08)	2.46 (2.30-2.62)	2.55 (2.37-2.74)
≥3	1.41 (1.26-1.56)	1.39 (1.32-1.47)	1.41 (1.33-1.50)	1.64 (1.57-1.71)	1.95 (1.84-2.06)	1.98 (1.87-2.11)
Calendar year of diagnosis						
2007-2009	1.79 (1.63-1.97)	1.71 (1.62-1.80)	1.89 (1.78-2.01)	2.54 (2.43-2.66)	3.18 (3.01-3.35)	3.24 (3.06-3.43)
2010-2012	1.61 (1.47-1.76)	1.56 (1.49-1.64)	1.70 (1.61-1.79)	2.34 (2.24-2.43)	3.05 (2.90-3.20)	3.16 (2.99-3.33)
2013-2015	1.56 (1.44-1.70)	1.42 (1.36-1.49)	1.62 (1.54-1.70)	2.29 (2.20-2.38)	2.94 (2.80-3.08)	3.08 (2.92-3.25)
2016-2017	1.60 (1.45-1.77)	1.45 (1.37-1.54)	1.70 (1.60-1.80)	2.47 (2.35-2.60)	3.04 (2.86-3.23)	3.17 (2.97-3.39)
Coresident status						
Cohabiting	1.59 (1.48-1.70)	1.49 (1.43-1.55)	1.74 (1.67-1.81)	2.51 (2.44-2.59)	3.25 (3.14-3.37)	3.42 (3.29-3.56)
Living alone	1.81 (1.71-1.91)	1.70 (1.65-1.76)	1.91 (1.85-1.97)	2.59 (2.52-2.65)	3.21 (3.11-3.31)	3.34 (3.23-3.46)
MMSE score^c^						
0-9	0.82 (0.47-1.43)	1.09 (0.79-1.50)	1.46 (1.04-2.04)	1.80 (1.41-2.30)	2.06 (1.54-2.76)	2.14 (1.53-2.98)
10-19	1.54 (1.33-1.78)	1.33 (1.22-1.44)	1.50 (1.37-1.64)	2.19 (2.05-2.35)	2.87 (2.64-3.11)	2.98 (2.73-3.26)
20-24	1.57 (1.38-1.79)	1.38 (1.29-1.49)	1.52 (1.41-1.64)	2.26 (2.14-2.40)	3.15 (2.93-3.37)	3.37 (3.11-3.64)
25-30	1.27 (1.08-1.50)	1.34 (1.23-1.47)	1.65 (1.51-1.81)	2.58 (2.40-2.77)	3.67 (3.37-4.00)	3.86 (3.51-4.25)
Type of diagnostic unit^c^						
Specialist care	1.51 (1.37-1.67)	1.41 (1.33-1.49)	1.67 (1.57-1.77)	2.67 (2.55-2.79)	3.95 (3.75-4.17)	4.19 (3.95-4.46)
Primary care	1.38 (1.21-1.57)	1.28 (1.19-1.38)	1.40 (1.30-1.51)	1.80 (1.69-1.91)	2.15 (2.00-2.32)	2.24 (2.07-2.44)

Abbreviations: HR, hazard ratio; MMSE, Mini-Mental State Examination.

^a^ All models were adjusted for age, sex, educational attainment (<9 years, 9-12 years, ≥13 years, missing), disposable individual income (in 100 SEK, approximately $9) (<1300, 1300 to <1562, 1562 to <1966, ≥1966), region of birth (Sweden or other), Charlson Comorbidity Index (0, 1, 2, ≥3, with higher values indicating more comorbidities), and calendar year of diagnosis (2007-2009, 2010-2012, 2013-2015, or 2016-2017).

^b^ Disease status within 3 years before the diagnosis of dementia disease.

^c^ Among patients from SveDem and matched controls; MMSE score categories, with higher scores indicating milder cognitive impairment.

**Table 2.** Hazard Ratios and 95% CIs of Psychiatric Disorders Among Patients With vs Those Without Dementia After Dementia Diagnosis, by Participant Characteristics^a^

Variables	Risk after diagnosis, HR (95% CI)								
	1 wk	0.5 y	1 y	2 y	3 y	5 y	6.5 y	8 y	10 y
Overall	4.74 (4.21-5.34)	2.00 (1.92-2.08)	1.59 (1.54-1.65)	1.26 (1.21-1.32)	1.10 (1.06-1.14)	0.93 (0.87-0.98)	0.85 (0.79-0.92)	0.79 (0.73-0.87)	0.74 (0.67-0.82)
Age at diagnosis, y									
<65	9.19 (5.16-16.35)	4.25 (3.53-5.12)	3.10 (2.68-3.58)	2.20 (1.87-2.60)	1.92 (1.65-2.24)	1.75 (1.49-2.05)	1.66 (1.33-2.07)	1.59 (1.22-2.08)	1.51 (1.11-2.07)
65-74	8.77 (6.47-11.90)	3.40 (3.07-3.77)	2.53 (2.33-2.75)	1.78 (1.61-1.98)	1.49 (1.36-1.63)	1.19 (1.06-1.33)	1.05 (0.90-1.24)	0.96 (0.79-1.17)	0.88 (0.70-1.09)
≥75	4.07 (3.56-4.66)	1.70 (1.62-1.78)	1.38 (1.33-1.45)	1.10 (1.05-1.16)	0.94 (0.90-0.98)	0.76 (0.71-0.82)	0.69 (0.62-0.76)	0.64 (0.57-0.71)	0.59 (0.52-0.67)
Sex									
Male	4.73 (3.94-5.66)	2.04 (1.92-2.18)	1.62 (1.53-1.71)	1.31 (1.23-1.40)	1.20 (1.13-1.27)	1.09 (0.99-1.19)	1.04 (0.92-1.16)	0.99 (0.87-1.14)	0.95 (0.82-1.11)
Female	4.73 (4.04-5.53)	1.95 (1.85-2.07)	1.57 (1.49-1.64)	1.22 (1.16-1.30)	1.04 (0.99-1.09)	0.83 (0.77-0.89)	0.74 (0.67-0.82)	0.68 (0.60-0.77)	0.62 (0.54-0.72)
Educational attainment, y									
<9	4.32 (3.56-5.24)	1.62 (1.52-1.74)	1.36 (1.28-1.44)	1.12 (1.05-1.20)	0.96 (0.90-1.02)	0.80 (0.73-0.88)	0.74 (0.65-0.83)	0.69 (0.60-0.80)	0.64 (0.55-0.76)
9-12	5.18 (4.31-6.22)	2.09 (1.96-2.23)	1.60 (1.51-1.70)	1.25 (1.17-1.34)	1.13 (1.07-1.20)	0.99 (0.90-1.08)	0.92 (0.82-1.03)	0.87 (0.76-1.00)	0.82 (0.70-0.96)
≥13	5.22 (3.95-6.89)	2.76 (2.50-3.06)	2.20 (2.02-2.40)	1.65 (1.49-1.83)	1.40 (1.28-1.53)	1.08 (0.94-1.24)	0.95 (0.79-1.14)	0.86 (0.69-1.08)	0.79 (0.61-1.01)
Charlson Comorbidity Index^b^									
0	6.87 (5.79-8.14)	2.52 (2.38-2.67)	1.93 (1.84-2.02)	1.44 (1.36-1.52)	1.23 (1.17-1.29)	1.02 (0.95-1.10)	0.93 (0.85-1.03)	0.87 (0.77-0.97)	0.80 (0.70-0.91)
1	3.95 (2.99-5.22)	1.54 (1.39-1.69)	1.23 (1.13-1.35)	0.99 (0.89-1.09)	0.87 (0.79-0.95)	0.72 (0.62-0.84)	0.66 (0.54-0.80)	0.62 (0.49-0.77)	0.57 (0.45-0.74)
2	3.47 (2.52-4.79)	1.73 (1.54-1.94)	1.39 (1.24-1.55)	1.13 (1.00-1.27)	1.01 (0.90-1.13)	0.89 (0.74-1.07)	0.83 (0.66-1.05)	0.79 (0.61-1.04)	0.75 (0.56-1.01)
≥ 3	2.57 (1.92-3.44)	1.38 (1.24-1.53)	1.20 (1.06-1.35)	1.03 (0.93-1.14)	0.90 (0.80-1.01)	0.75 (0.61-0.92)	0.68 (0.53-0.89)	0.64 (0.48-0.86)	0.61 (0.44-0.84)
Calendar year of diagnosis									
2007-2009	5.13 (4.13-6.37)	1.98 (1.83-2.15)	1.52 (1.43-1.63)	1.15 (1.06-1.25)	1.04 (0.96-1.11)	0.95 (0.88-1.02)	0.89 (0.80-0.99)	0.85 (0.74-0.96)	0.80 (0.68-0.93)
2010-2012	4.46 (3.57-5.58)	1.90 (1.76-2.05)	1.53 (1.44-1.63)	1.21 (1.12-1.31)	1.06 (0.99-1.13)	0.87 (0.79-0.97)	0.80 (0.69-0.91)	0.75 (0.64-0.87)	0.70 (0.59-0.83)
2013-2015	5.04 (3.99-6.36)	2.09 (1.94-2.26)	1.80 (1.66-1.95)	1.42 (1.32-1.52)	1.18 (1.06-1.31)	0.97 (0.82-1.15)	0.89 (0.74-1.08)	0.83 (0.68-1.02)	0.78 (0.62-0.97)
2016-2017	4.19 (3.03-5.78)	2.23 (1.88-2.66)	1.60 (1.36-1.89)	1.20 (0.89-1.63)	1.04 (0.72-1.50)	0.86 (0.55-1.35)	0.78 (0.48-1.28)	0.73 (0.43-1.23)	0.67 (0.38-1.17)
Coresident									
Cohabiting	4.54 (3.83-5.38)	2.30 (2.17-2.44)	1.84 (1.75-1.93)	1.45 (1.36-1.53)	1.23 (1.17-1.30)	1.02 (0.94-1.11)	0.94 (0.84-1.04)	0.87 (0.77-0.99)	0.81 (0.70-0.94)
Living alone	5.07 (4.41-5.83)	1.84 (1.74-1.94)	1.45 (1.39-1.52)	1.16 (1.10-1.23)	1.03 (0.98-1.08)	0.88 (0.81-0.95)	0.81 (0.73-0.90)	0.76 (0.67-0.86)	0.71 (0.62-0.82)
MMSE score^c^									
0-9	2.52 (1.10-5.79)	1.93 (1.30-2.89)	1.60 (1.01-2.53)	1.13 (0.70-1.83)	0.90 (0.57-1.42)	0.66 (0.28-1.52)	0.56 (0.19-1.65)	0.50 (0.14-1.71)	0.44 (0.11-1.73)
10-19	3.31 (2.23-4.92)	1.97 (1.73-2.24)	1.44 (1.28-1.61)	1.01 (0.88-1.16)	0.86 (0.76-0.98)	0.66 (0.52-0.83)	0.58 (0.43-0.78)	0.53 (0.37-0.75)	0.48 (0.32-0.71)
20-24	3.88 (2.73-5.51)	2.11 (1.89-2.36)	1.86 (1.70-2.03)	1.52 (1.37-1.69)	1.21 (1.10-1.33)	0.87 (0.74-1.01)	0.74 (0.60-0.91)	0.66 (0.51-0.85)	0.59 (0.45-0.78)
25-30	4.08 (2.48-6.71)	2.52 (2.19-2.89)	2.02 (1.82-2.24)	1.56 (1.37-1.77)	1.43 (1.29-1.59)	1.24 (1.06-1.44)	1.15 (0.93-1.41)	1.09 (0.85-1.39)	1.03 (0.78-1.36)
Type of diagnostic unit^c^									
Specialist care	4.83 (3.71-6.29)	2.72 (2.50-2.96)	2.10 (1.96-2.26)	1.53 (1.40-1.66)	1.28 (1.19-1.38)	0.98 (0.87-1.10)	0.85 (0.73-1.00)	0.77 (0.64-0.93)	0.70 (0.57-0.87)
Primary care	2.58 (1.80-3.69)	1.52 (1.36-1.70)	1.37 (1.24-1.50)	1.19 (1.07-1.32)	1.04 (0.95-1.15)	0.88 (0.75-1.04)	0.82 (0.67-1.00)	0.77 (0.61-0.97)	0.73 (0.56-0.95)

Abbreviations: HR, hazard ratio; MMSE, Mini-Mental State Examination.

^a^ All models were adjusted for age, sex, educational attainment (<9 years, 9-12 years, ≥13 years, missing), disposable individual income (in 100 SEK, approximately $9) (<1300, 1300 to <1562, 1562 to <1966, ≥1966), region of birth (Sweden or other), Charlson Comorbidity Index (0, 1, 2, ≥3, with higher values indicating more comorbidities), and calendar year of diagnosis (2007-2009, 2010-2012, 2013-2015, or 2016-2017).

^b^ Disease status within 3 years before the diagnosis of dementia disease.

^c^ Among patients from SveDem and matched controls; MMSE score categories, with higher scores indicating milder cognitive impairment.

**Figure 2.**
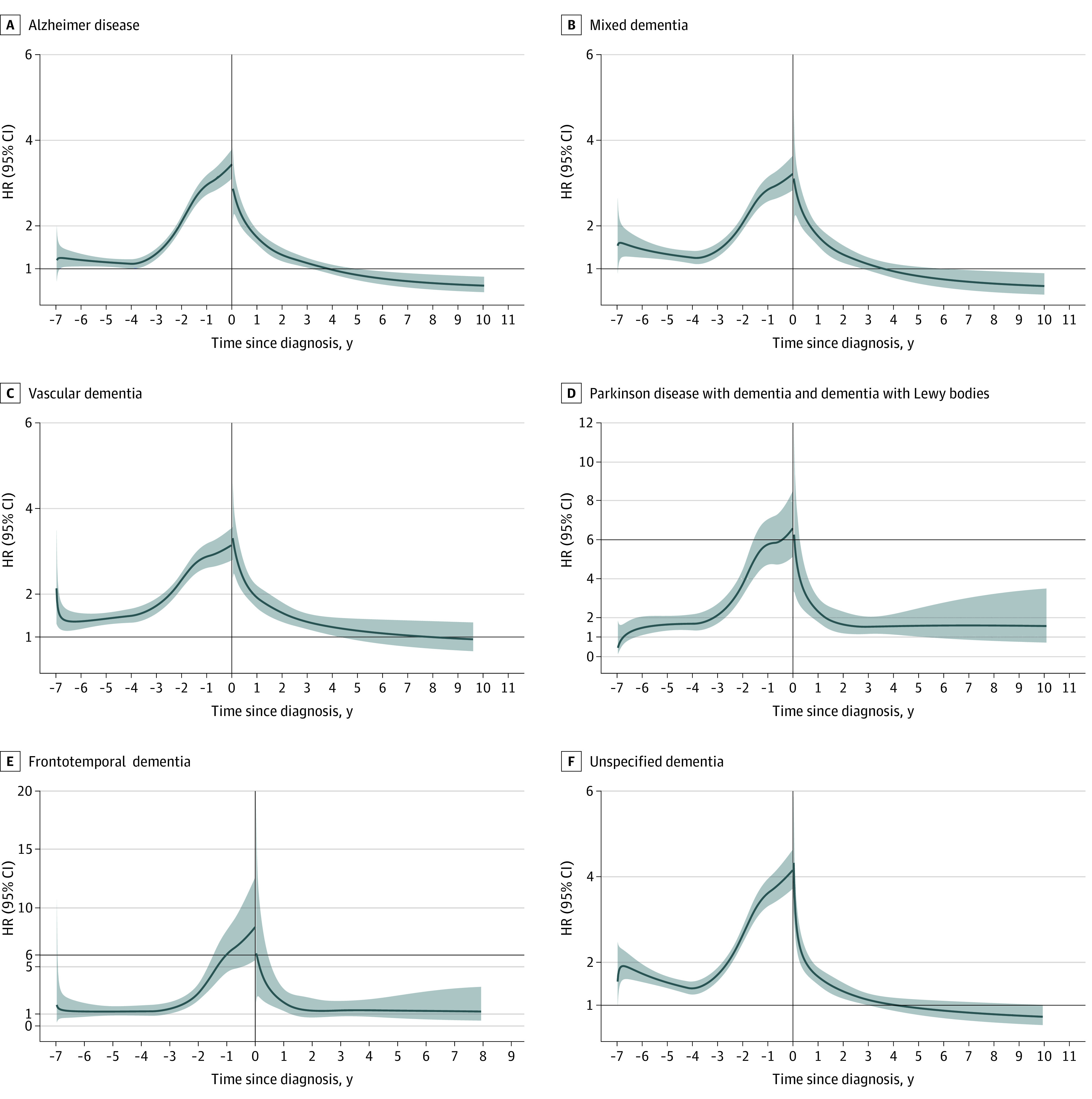
Hazard Ratios (HRs) and 95% CIs of Psychiatric Disorders Before and After Dementia Diagnosis in a Matched Cohort Study, by Dementia Types in Sweden, 2000 to 2017

The results were largely similar for AD, mixed dementia, vascular dementia and unspecified dementia (Figure 2). The HRs were greater for LBD and FTD from 2 years before diagnosis (LBD HR, 3.72 [95% CI, 3.11-4.45]; and FTD HR, 2.78 [95% CI, 2.17-3.56]) to 3 years after diagnosis (LBD HR, 1.31 [95% CI, 0.81-2.11]; and FTD HR, 1.13 [95% CI, 1.00-1.27]) compared with other dementia subtypes (eTable 9 and eTable 10 in Supplement 1).

We also observed similar results for individual psychiatric disorders, except sleep disorders and somatoform/conversion disorders (eFigure 5 in Supplement 1). Stress-related disorders and psychotic disorders exhibited the most substantial rate increase immediately following dementia diagnosis.

**Figure 3.**
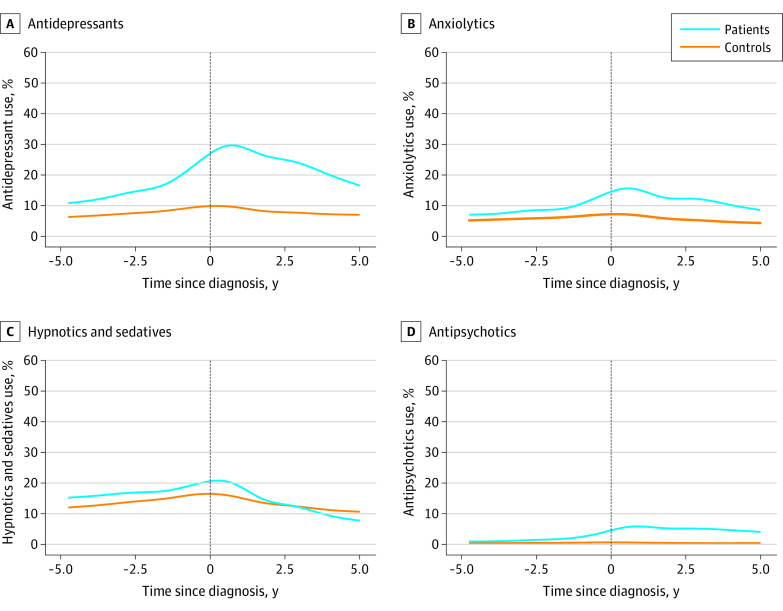
Use of Antidepressants, Anxiolytics, Hypnotics and Sedatives, and Antipsychotics Before and After Dementia Diagnosis in a Matched Cohort Study in Sweden, 2006 to 2017

In the subgroup analysis, we also found that the use of antidepressants was persistently higher among patients with dementia compared with controls, and the difference increased from 2 years before dementia diagnosis (15.9% vs 7.9%, *P* < .001), peaked approximately 6 months after dementia diagnosis (29.1% vs 9.7%, *P* < .001), and then decreased slowly from 3 years after diagnosis but remained higher than controls 5 years after diagnosis (16.4% vs 6.9%, *P* < .001) (Figure 3 and eTable 11 in Supplement 1). For anxiolytics and antipsychotics, the increased use was observed 2 years (8.8% vs 6.0%, *P* < <.001) and 1 year (2.3% vs 0.5%, *P* < .001) before dementia diagnosis and remained elevated thereafter. The use of hypnotics or sedatives was persistently higher among patients with dementia compared with controls before diagnosis, with the increased difference starting 6 months before diagnosis (18.5% vs 16.1%, *P* < .001) and decreasing slowly thereafter. A reversed pattern was observed 4 years after diagnosis (9.5% vs 11.2%, *P* < .001). Similar results were noted for dementia subtypes (eFigures 6, 7, 8, and 9 in Supplement 1).

## Discussion

In this nationwide population-based cohort study, we found that compared with participants without dementia, new-onset psychiatric disorders were more common among patients with dementia both before and after receipt of a diagnosis, especially among patients diagnosed as having LBD and FTD. Patients with dementia had greatly increased risks of almost all psychiatric disorders assessed, including depression, anxiety, stress-related disorders, substance use disorders, and psychotic disorders, as early as 7 years prior to diagnosis. Similar patterns were observed in AD, mixed dementia, vascular dementia, and unspecified dementia. Risk factors associated with psychiatric disorders among dementia patients were being younger, being better educated, receiving a diagnosis in specialist care, and having fewer physical comorbidities or higher MMSE score.

It is widely acknowledged that individuals with dementia are more likely to experience depression and other psychiatric disorders.^10,11,12,28^ Several psychiatric disorders have long been associated with cognitive impairment, and there is a growing recognition that cognitive deficits are not solely a consequence of mood disturbances in depression but may persist even after clinical recovery.^29^ The severity and persistence of enduring deficits have also been emphasized.^30^ Furthermore, depression has been identified as a risk factor for future dementia, and this association cannot be attributed solely to misdiagnosis of early cases of dementia.^11^ The association persists even for individuals who exhibited depressive symptoms for longer than 2 decades (28 years) prior to the onset of dementia.^11^

New psychiatric symptoms after the dementia diagnosis, known as neuropsychiatric symptoms, are prevalent among people with AD and related dementias and are associated with the course of disease.^31^ Previous studies have found that neuropsychiatric symptoms are mostly attributed to dementia symptomatology, which makes looking for new psychiatric diagnoses in a patient with dementia less relevant.^17,32^ This finding was also confirmed in our study by the high prevalence of psychiatric symptoms during the dementia disease progression and for all dementia disorders. However, it is necessary to differentiate generalized from disorder-specific risk factors over the disease course by evaluating the full range of psychiatric conditions prospectively.

We also found that patients with LBD and FTD had higher risk of psychiatric comorbidities compared with other dementia subtypes, which is in line with previous studies.^33,34^ These findings suggested that observing neuropsychiatric symptoms could be in favor of diagnoses of LBD and FTD. The high occurrence of psychiatric disorders and the difference across dementia subtypes highlight the importance of reducing the psychiatric health burden among patients with all dementia subtypes.

To our knowledge, our study constitutes the most comprehensive endeavor to date to quantify associations and to identify temporal patterns of new onset or diagnosis of psychiatric conditions among individuals with dementia before, during, and after diagnosis receipt. Our finding of the time-dependent risk pattern is in line with previous studies focusing on health care costs of dementia and injurious falls.^9,35,36^ A Swedish population-based study reported that patients with dementia exhibited an elevated occurrence of injurious falls, with the highest incidence observed 4 years prior to the diagnosis, peaking in the year of diagnosis, and declining rapidly during the 4 years after diagnosis.^36^ A case-control study in Germany also found an increase in the use of ambulatory medical care services by patients with dementia of 50% in the year before and of 40% in the year after the incidence, predominantly in primary care and neurology or psychiatry settings.^35^ Our finding of significant rise in risk observed immediately following a dementia diagnosis and decreased risk several years after diagnosis is consistent with earlier studies that have reported a substantially higher risk of suicide immediately following a dementia diagnosis and a lower risk thereafter.^6,37^ The markedly elevated risks observed in the year leading up to the diagnosis may be associated with the pre-existing symptoms of dementia and the significant psychological burden of receiving clinical assessment for a suspected disease.^6,37^

### Strengths and Limitations

This study has several strengths, including a large and national sample, a population-based cohort design, long follow-up, and a range of different types of dementia. Furthermore, sociodemographic characteristics, medical disorders, specific dementia subtypes, characteristics related to dementia, and psychiatric medications were also explored.

This study has limitations. The diagnoses of different types of dementia were only available for patients from SveDem. According to incidence estimates, SveDem captured almost one-third of all expected new dementia cases in Sweden. However, assessing the accuracy of these diagnoses is difficult, although they are consistent with standard clinical practice and comply with national guidelines and do not change with follow-up.^38^ Furthermore, the assessment of the study’s outcomes is based on clinical diagnoses obtained through specialized inpatient and outpatient care, which primarily represents more severe manifestations of the studied psychiatric disorders. In Sweden, first-line psychiatric care is administered through primary care, for which we did not have data. However, including psychiatric medication prescriptions (which should have almost complete coverage) was our attempt to compensate for this. The comparable temporal patterns observed in the augmented use of psychiatric medications (although with a more pronounced effect) confirm the high prevalence of psychiatric symptoms during dementia disease progression and suggest that the diagnosis of dementia may be associated with both severe and mild psychiatric conditions. Therefore, the combined use of both clinical diagnoses and medication use may capture the overall health burden during the period of diagnosis. However, the validity of using data on prescriptions for psychiatric medications as a surrogate diagnostic marker of the underlying diagnoses needs to be further explored. In addition, co-occurring psychiatric disorders are common among patients with dementia. Patients who develop early dementia or psychiatric illness symptoms or are diagnosed formally with these illnesses will contact medical settings more frequently and thus be more likely to receive a diagnosis of concurrent partner illnesses. The extent to which this possibility may be associated with our findings remains uncertain.

## Conclusions

In this cohort study, patients with dementia had markedly increased risks of receiving a diagnosis of a psychiatric disorder and a prescription for psychiatric medications both before and after dementia diagnosis receipt. These findings highlight the significance of incorporating psychiatric preventative and management interventions for individuals with dementia across various stages of the diagnosis process and confirm the importance of management of psychiatric symptoms during the dementia disease progression.

## Notes

Supplement 1.
eMethods.
**eFigure 1.** Flowchart of study patients with dementia and control participants **eFigure 2.** Cumulative incidences and 95% confidence intervals of individual psychiatric disorders between people with and without dementia before and after dementia diagnosis in the study population in Sweden, 2000 to 2017 **eFigure 3**. Hazard ratios and 95% CIs of individual psychiatric disorders before and after dementia diagnosis in patients from SveDem and matched controls, 2000 to 2017 **eFigure 4.** Hazard ratios and 95% CIs of individual psychiatric disorders before and after dementia diagnosis in patients from other registers and matched controls, 2000 to 2017 **eFigure 5.** Hazard ratios and 95% CIs of individual psychiatric disorders before and after dementia diagnosis in a matched cohort study in Sweden, 2000 to 2017 **eFigure 6.** Use of antidepressants by dementia types before and after a specific dementia diagnosis in patients from SveDem and matched controls, 2006 to 2017 **eFigure 7.** Use of anxiolytics by dementia types before and after a specific dementia diagnosis in patients from SveDem and matched controls, 2006 to 2017 **eFigure 8.** Use of hypnotics/sedatives by dementia types before and after a specific dementia diagnosis in patients from SveDem and matched controls, 2006 to 2017 **eFigure 9.** Use of antipsychotics by dementia types before and after a specific dementia diagnosis in patients from SveDem and matched controls, 2006 to 2017 **eTable 1.** ICD-10 and ATC codes of inclusion criteria for patients with dementia from the National Patient Register and the Swedish Prescribed Drug Register, and exclusion criteria for dementia-free subjects **eTable 2.** ICD-10 codes for psychiatric disorders **eTable 3.** ATC codes for psychiatric medications **eTable 4.** Characteristics of dementia patients and the control participants at the index date **eTable 5.** Distribution of psychiatric disorders, person-years and incidence rates in a matched cohort study in Sweden, 2000 to 2017 **eTable 6.** Distribution of psychiatric disorders, person-years and incidence rates in patients from SveDem and matched controls, 2000 to 2017 **eTable 7.** Distribution of psychiatric disorders, person-years and incidence rates in patients from other registers and matched controls, 2000 to 2017 **eTable 8.** Distribution of psychiatric disorders, person-years and incidence rates by a specific dementia diagnosis in patients from SveDem and matched controls, 2000 to 2017 **eTable 9.** Hazard ratios and 95% CIs of psychiatric disorders before a specific dementia diagnosis in patients from SveDem and matched controls, 2000 to 2017^a^ **eTable 10.** Hazard ratios and 95% CIs of psychiatric disorders after a specific dementia diagnosis in patients from SveDem and matched controls, 2000 to 2017^a^ **eTable 11.** Use of antidepressants, anxiolytics, hypnotics/sedatives and antipsychotics before and after dementia diagnosis in a matched cohort study in Sweden, 2006 to 2017^a^

Supplement 2.
Data Sharing Statement
