# Supplementary material for: Psychiatric Disorders Before and After Dementia Diagnosis
Source: JAMA Netw Open. 2023 Oct 17;6(10):e2338080. doi: 10.1001/jamanetworkopen.2023.38080 (PMC10582787; doi:10.1001/jamanetworkopen.2023.38080)
Supplement: Supplement 1. — eMethods. eFigure 1. Flowchart of study patients with dementia and control participants eFigure 2. Cumulative incidences and 95% confidence intervals of individual psychiatric disorders between people with and without dementia before and after dementia diagnosis in the study population in Sweden, 2000 to 2017 eFigure 3. Hazard ratios and 95% CIs of individual psychiatric disorders before and after dementia diagnosis in patients from SveDem and matched controls, 2000 to 2017 eFigure 4. Hazard ratios and 95% CIs of individual psychiatric disorders before and after dementia diagnosis in patients from other registers and matched controls, 2000 to 2017 eFigure 5. Hazard ratios and 95% CIs of individual psychiatric disorders before and after dementia diagnosis in a matched cohort study in Sweden, 2000 to 2017 eFigure 6. Use of antidepressants by dementia types before and after a specific dementia diagnosis in patients from SveDem and matched controls, 2006 to 2017 eFigure 7. Use of anxiolytics by dementia types before and after a specific dementia diagnosis in patients from SveDem and matched controls, 2006 to 2017 eFigure 8. Use of hypnotics/sedatives by dementia types before and after a specific dementia diagnosis in patients from SveDem and matched controls, 2006 to 2017 eFigure 9. Use of antipsychotics by dementia types before and after a specific dementia diagnosis in patients from SveDem and matched controls, 2006 to 2017 eTable 1. ICD-10 and ATC codes of inclusion criteria for patients with dementia from the National Patient Register and the Swedish Prescribed Drug Register, and exclusion criteria for dementia-free subjects eTable 2. ICD-10 codes for psychiatric disorders eTable 3. ATC codes for psychiatric medications eTable 4. Characteristics of dementia patients and the control participants at the index date eTable 5. Distribution of psychiatric disorders, person-years and incidence rates in a matched cohort study in Sweden, 2000 to 2017 eTable 6. Distribution o [file jamanetwopen-e2338080-s001.pdf]

Supplementary Online Content

Mo M, Zacarias-Pons L, Hoang MT, et al. Psychiatric disorders before and after dementia diagnosis. *JAMA Netw Open*. 2023;6(10):e2338080. doi:10.1001/jamanetworkopen.2023.38080

eMethods.

eFigure 1. Flowchart of study patients with dementia and control participants

eFigure 2. Cumulative incidences and 95% confidence intervals of individual psychiatric disorders between people with and without dementia before and after dementia diagnosis in the study population in Sweden, 2000 to 2017

eFigure 3. Hazard ratios and 95% CIs of individual psychiatric disorders before and after dementia diagnosis in patients from SveDem and matched controls, 2000 to 2017

eFigure 4. Hazard ratios and 95% CIs of individual psychiatric disorders before and after dementia diagnosis in patients from other registers and matched controls, 2000 to 2017

eFigure 5. Hazard ratios and 95% CIs of individual psychiatric disorders before and after dementia diagnosis in a matched cohort study in Sweden, 2000 to 2017

eFigure 6. Use of antidepressants by dementia types before and after a specific dementia diagnosis in patients from SveDem and matched controls, 2006 to 2017

eFigure 7. Use of anxiolytics by dementia types before and after a specific dementia diagnosis in patients from SveDem and matched controls, 2006 to 2017

eFigure 8. Use of hypnotics/sedatives by dementia types before and after a specific dementia diagnosis in patients from SveDem and matched controls, 2006 to 2017

eFigure 9. Use of antipsychotics by dementia types before and after a specific dementia diagnosis in patients from SveDem and matched controls, 2006 to 2017

eTable 1. ICD-10 and ATC codes of inclusion criteria for patients with dementia from the National Patient Register and the Swedish Prescribed Drug Register, and exclusion criteria for dementia-free subjects

eTable 2. ICD-10 codes for psychiatric disorders

eTable 3. ATC codes for psychiatric medications

eTable 4. Characteristics of dementia patients and the control participants at the index date

© 2023 last Mo M et al. *JAMA Network Open*.

eTable 5. Distribution of psychiatric disorders, person-years and incidence rates in a matched cohort study in Sweden, 2000 to 2017

eTable 6. Distribution of psychiatric disorders, person-years and incidence rates in patients from SveDem and matched controls, 2000 to 2017

eTable 7. Distribution of psychiatric disorders, person-years and incidence rates in patients from other registers and matched controls, 2000 to 2017

eTable 8. Distribution of psychiatric disorders, person-years and incidence rates by a specific dementia diagnosis in patients from SveDem and matched controls, 2000 to 2017

eTable 9. Hazard ratios and 95% CIs of psychiatric disorders before a specific dementia diagnosis in patients from SveDem and matched controls, 2000 to 2017[a]

eTable 10. Hazard ratios and 95% CIs of psychiatric disorders after a specific dementia diagnosis in patients from SveDem and matched controls, 2000 to 2017[a]

eTable 11. Use of antidepressants, anxiolytics, hypnotics/sedatives and antipsychotics before and after dementia diagnosis in a matched cohort study in Sweden, 2006 to 2017[a]

eReferences

This supplementary material has been provided by the authors to give readers additional information about their work.

© 2023 last Mo M et al. *JAMA Network Open*.

eMethods

Register database

SveDem is a quality registry that was initiated in 2007 to monitor and improve the quality of diagnostic work-up, treatment and care of dementia patients in Sweden. It includes patients with incident dementia diagnoses from either primary care or specialist memory clinics and contains information on diagnostic variables (type of dementia, MMSE scores at the time of dementia diagnosis) [1]. The National Patient Register contains nationwide records on inpatient care since 1987 and more than 80% of specialized (hospital-based) outpatient care since 2001 with high validity (85-95%) [2]. The Swedish Prescribed Drug Register provides complete data on dispensation of prescription medications from all pharmacies since July 2005 [3]. Data on the number of individuals exposed to certain drugs may also be used as a proxy to estimate the prevalence of diseases and the drug utilization could also be compared to the prevalence of a disease as a rough measurement of the appropriateness of prescribing [3]. Together, the National Patient Register and the Prescribed Drug Register cover >99% of all inpatient medical diagnoses and expedited drugs [2,3]. The Swedish unique personal identification number was used by Swedish authorities and register holders to identify patients across sources and to merge data.

Subgroup analysis

Antidepressants, antipsychotics (excluding clozapine [4,5]), anxiolytics, and hypnotics/sedatives (for ATC codes, see eTable 3) were identified through the Swedish Prescribed Drug Register. Clozapine was not included because it is licensed only for patients with treatment-resistant psychosis (shown insufficient response to treatment) [4] and necessitates regular blood testing, which might result in a selected group with prescription of this drug, and its efficacy is significantly higher than other antipsychotics [5]. We included patients with dementia (n= 40 457) and matched control participants (n= 107 893) with an index date during 2011-2012 in this analysis and followed them from 5 years before the index date (earliest possible entry on January 1, 2006) to death, December 31, 2017, or 5 years after the index date, whichever came first. In Sweden, the guideline is to dispense up to 3 months of medications at a time [6] in routine clinical practice, so we considered patients treated during the 3 months following each dispensation. A patient was defined as receiving antipsychotics during 14 days after each dispensation of depot antipsychotic[6].

© 2023 Mo M et al. *JAMA Network Open*.

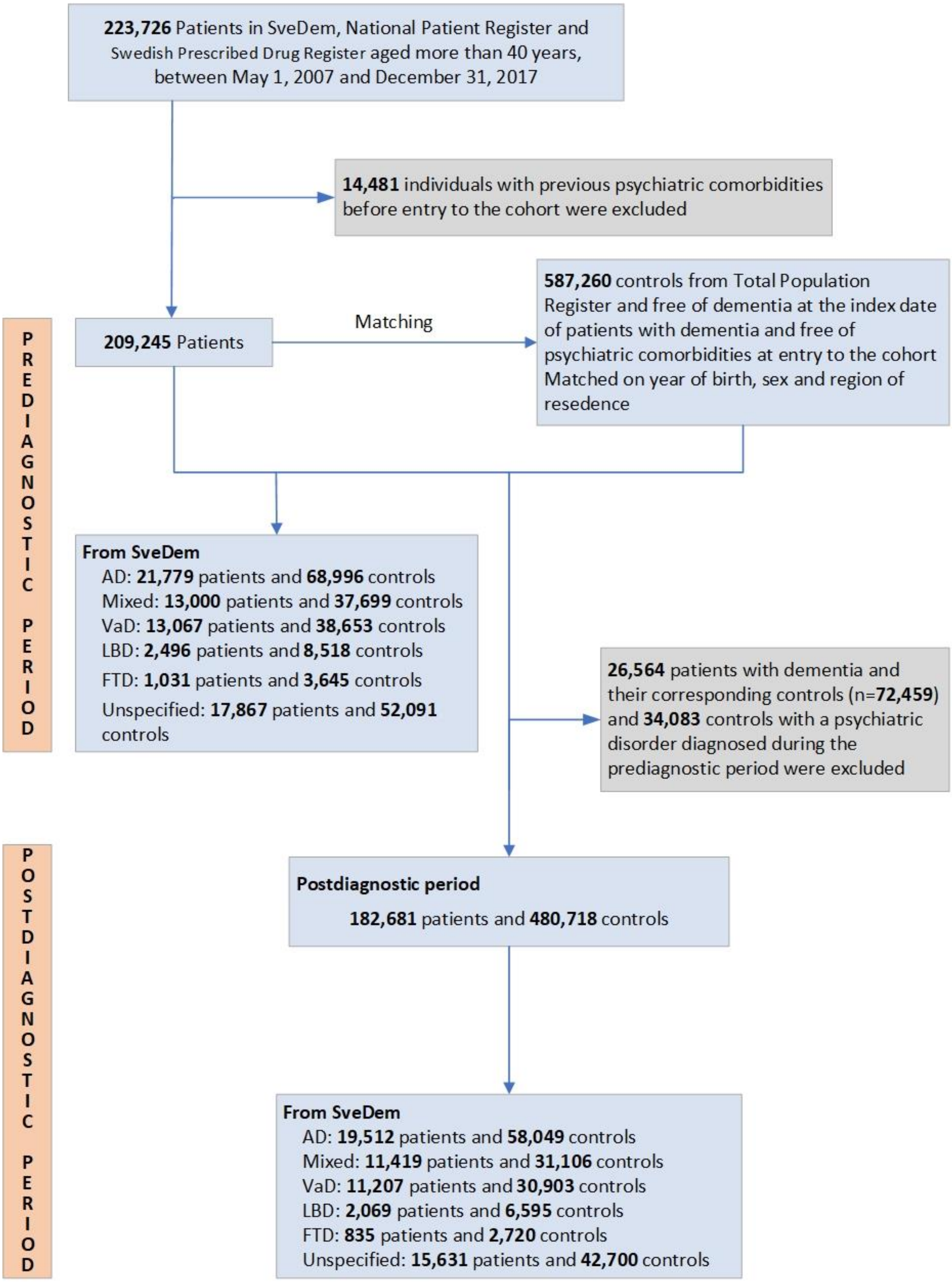

eFigure 1. Flowchart of study patients with dementia and control participants

Abbreviations: SveDem, the Swedish Registry for Cognitive/Dementia Disorders; AD, Alzheimer's disease; Mixed, mixed dementia; VaD, vascular dementia; LBD, Parkinson's disease with dementia and dementia with Lewy bodies; FTD, frontotemporal dementia; Unspecified, unspecified dementia.

© 2023 Mo M et al. *JAMA Network Open*.

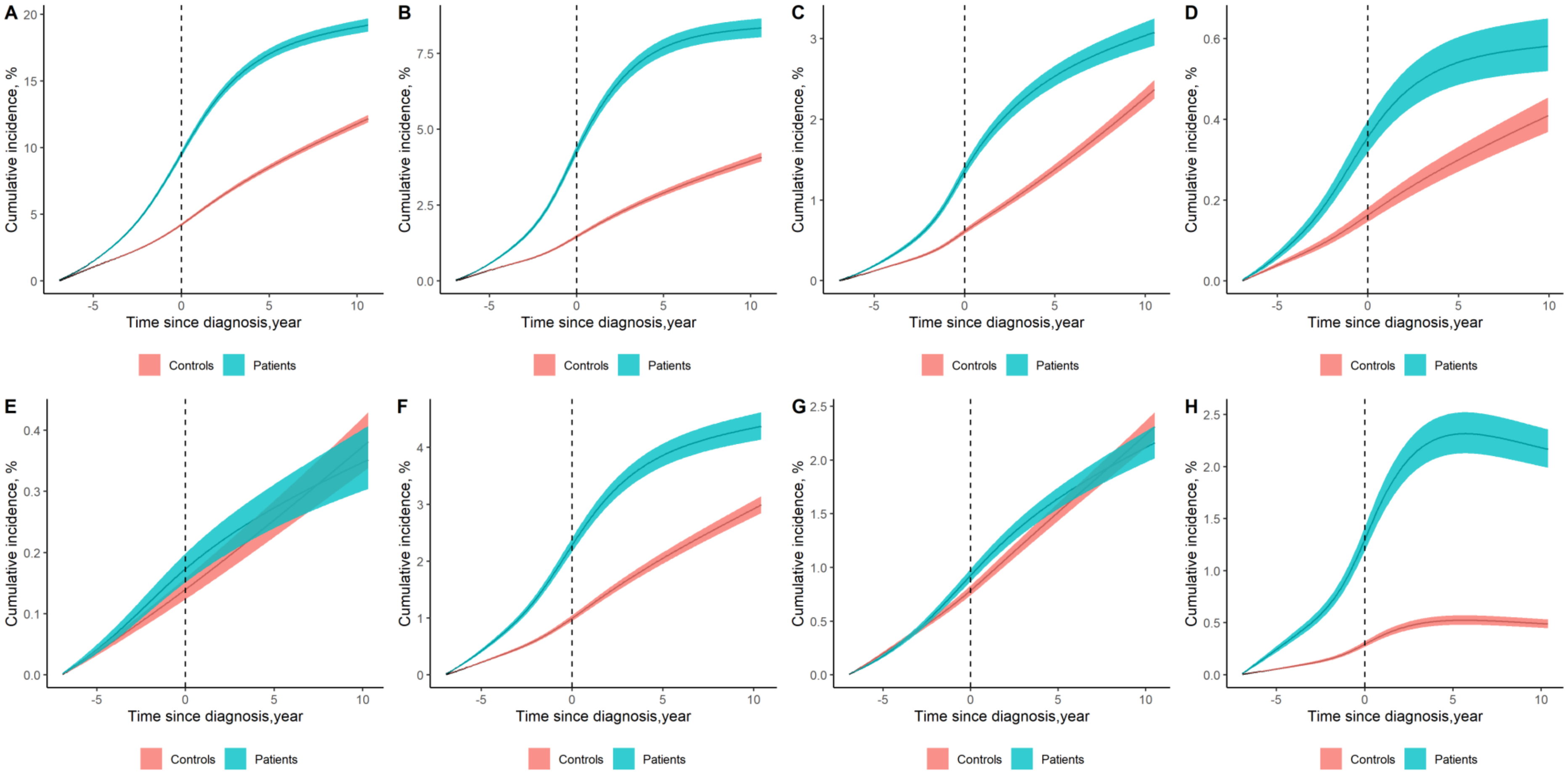

eFigure 2. Cumulative incidences and 95% confidence intervals of individual psychiatric disorders between people with and without dementia before and after dementia diagnosis in the study population in Sweden, 2000 to 2017

A. Overall, B. Depression, C. Anxiety, D. Stress-related disorders, E. Somatoform/conversion disorders, F. Substance abuse disorders, G. Sleep disorders, H. Psychotic disorders.

© 2023 Mo M et al. *JAMA Network Open*.

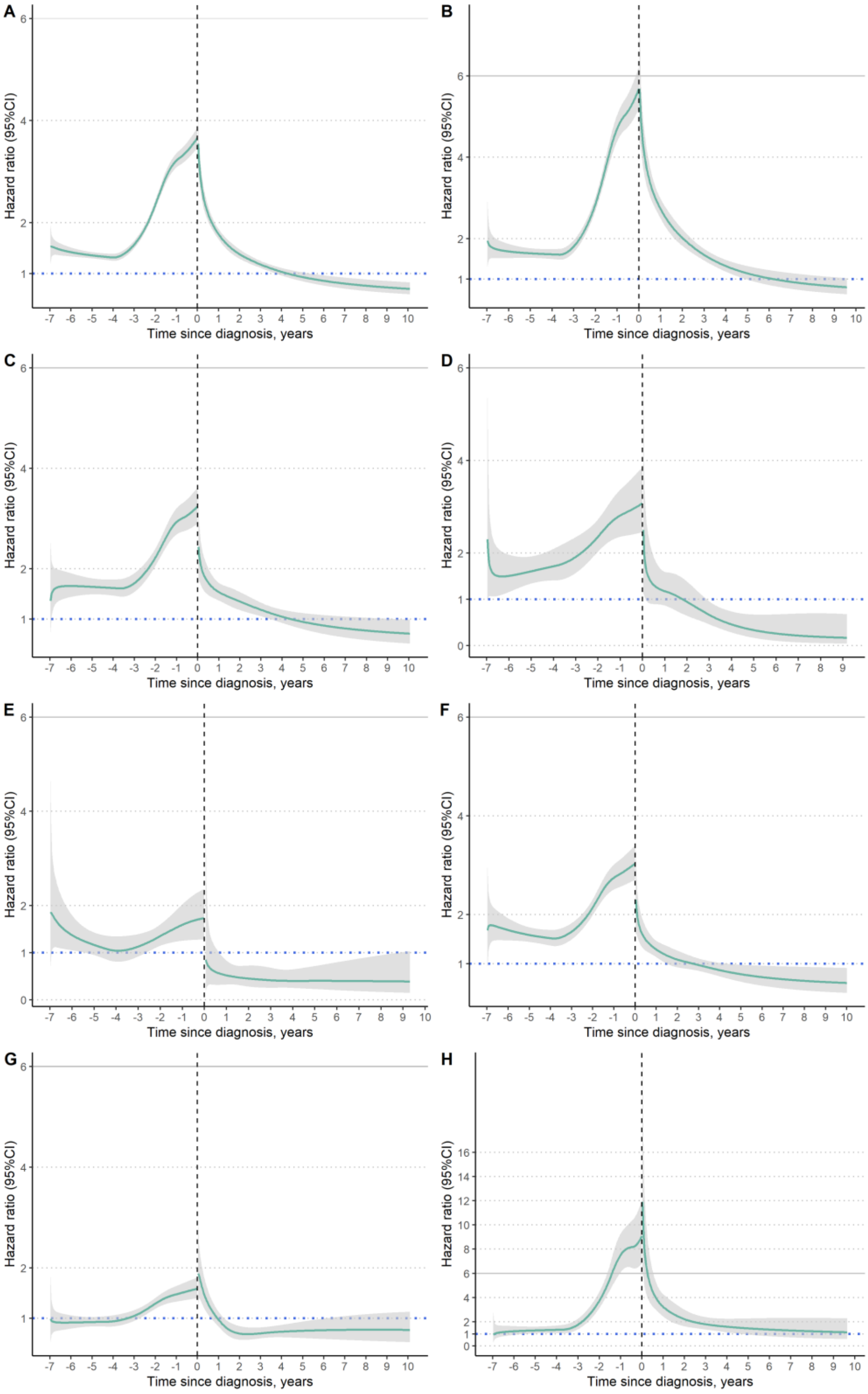

eFigure 3. Hazard ratios and 95% CIs of individual psychiatric disorders before and after dementia diagnosis in patients from SveDem and matched controls, 2000 to 2017

A. Overall, B. Depression, C. Anxiety, D. Stress-related disorders, E. Somatoform/conversion disorders, F. Substance abuse disorders, G. Sleep disorders, H. Psychotic disorders.

© 2023 Mo M et al. *JAMA Network Open*.

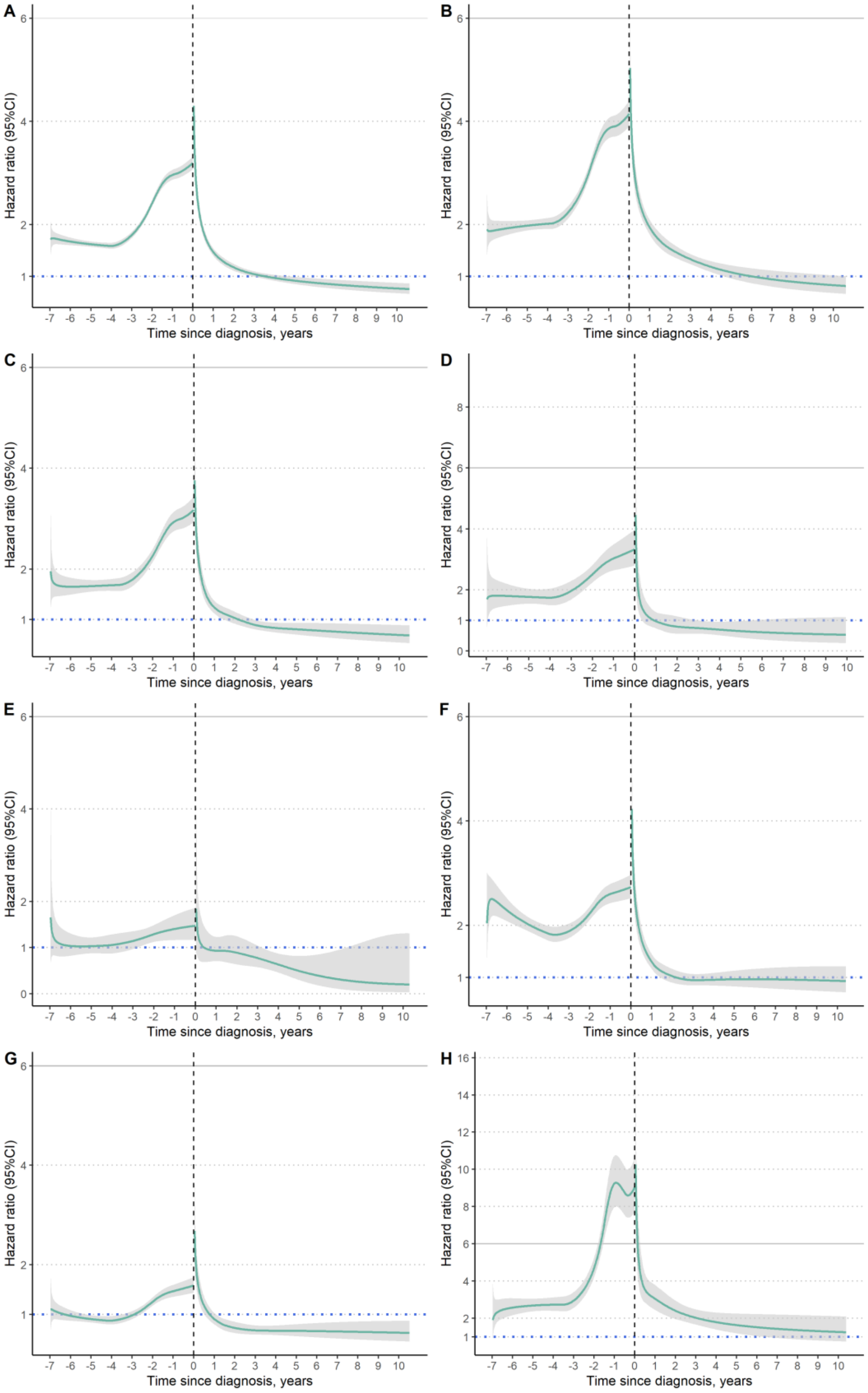

eFigure 4. Hazard ratios and 95% CIs of individual psychiatric disorders before and after dementia diagnosis in patients from other registers and matched controls, 2000 to 2017

A. Overall, B. Depression, C. Anxiety, D. Stress-related disorders, E. Somatoform/conversion disorders, F. Substance abuse disorders, G. Sleep disorders, H. Psychotic disorders.

© 2023 Mo M et al. *JAMA Network Open*.

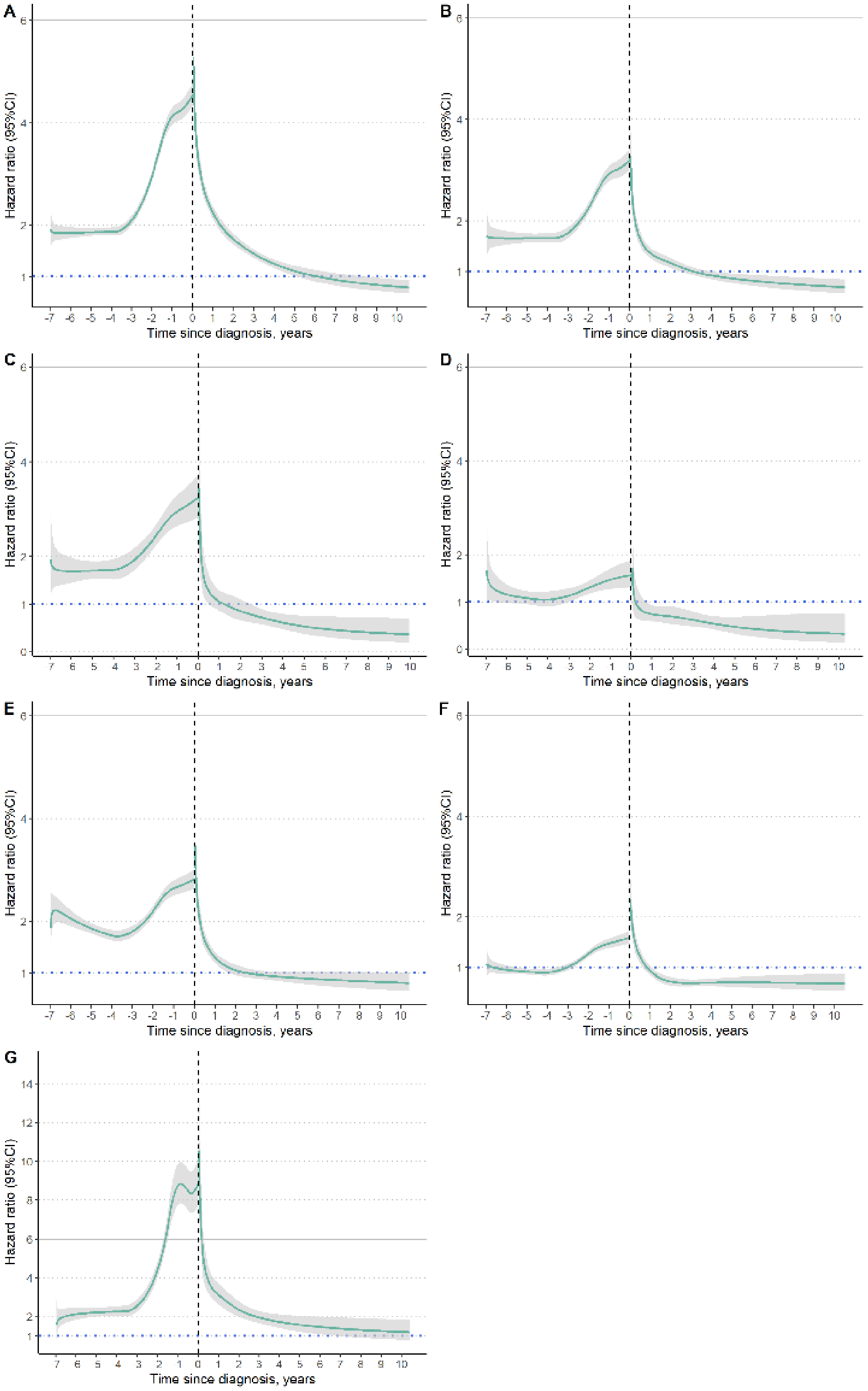

eFigure 5. Hazard ratios and 95% CIs of individual psychiatric disorders before and after dementia diagnosis in a matched cohort study in Sweden, 2000 to 2017

A. Depression, B. Anxiety, C. Stress-related disorders, D. Somatoform/conversion disorders, E. Substance abuse disorders, F. Sleep disorders, G. Psychotic disorders.

© 2023 Mo M et al. *JAMA Network Open*.

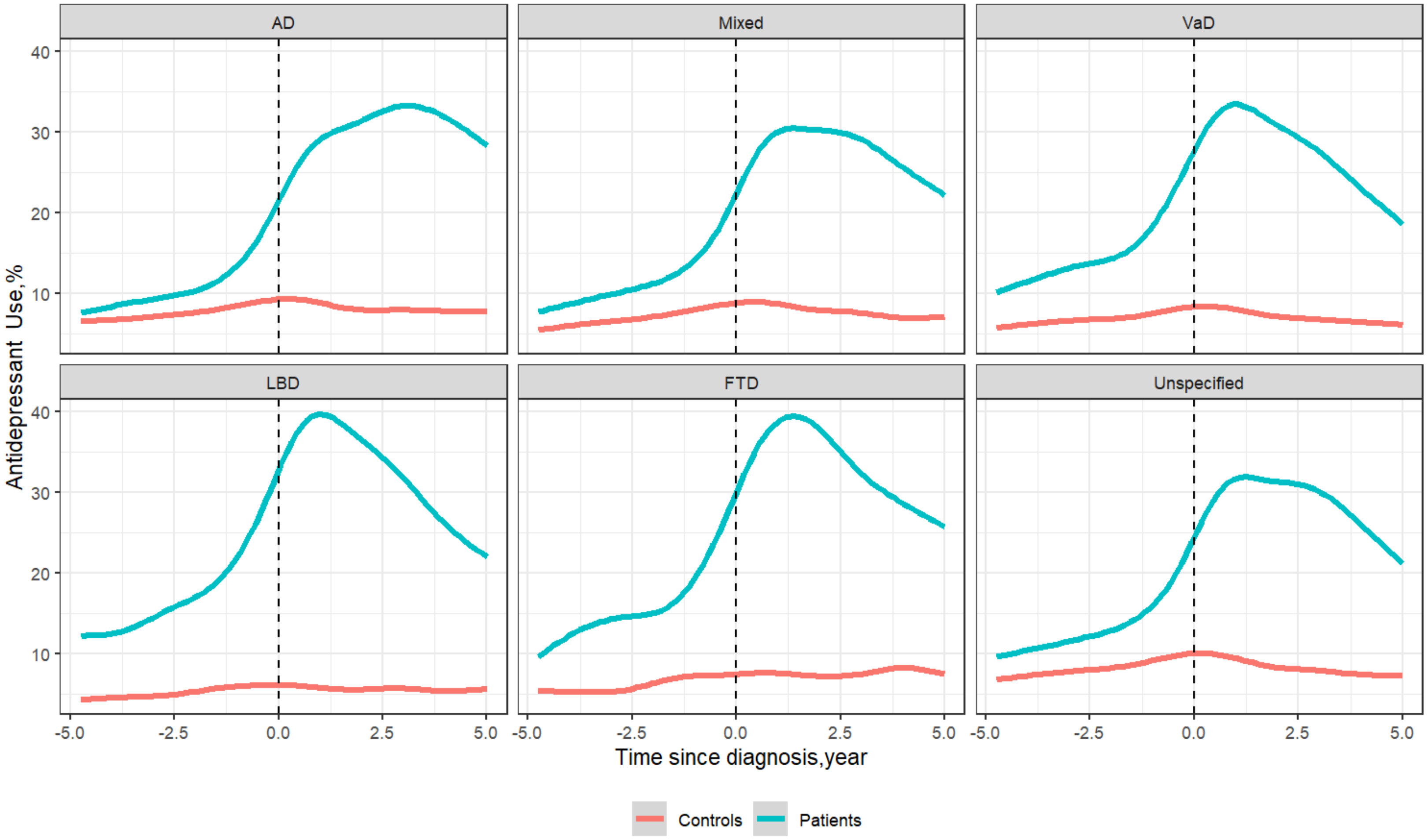

eFigure 6. Use of antidepressants by dementia types before and after a specific dementia diagnosis in patients from SveDem and matched controls, 2006 to 2017

Abbreviations: AD, Alzheimer's disease; Mixed, mixed dementia; VaD, vascular dementia; LBD, Parkinson's disease with dementia and dementia with Lewy bodies; FTD, frontotemporal dementia; Unspecified, unspecified dementia.

© 2023 Mo M et al. *JAMA Network Open*.

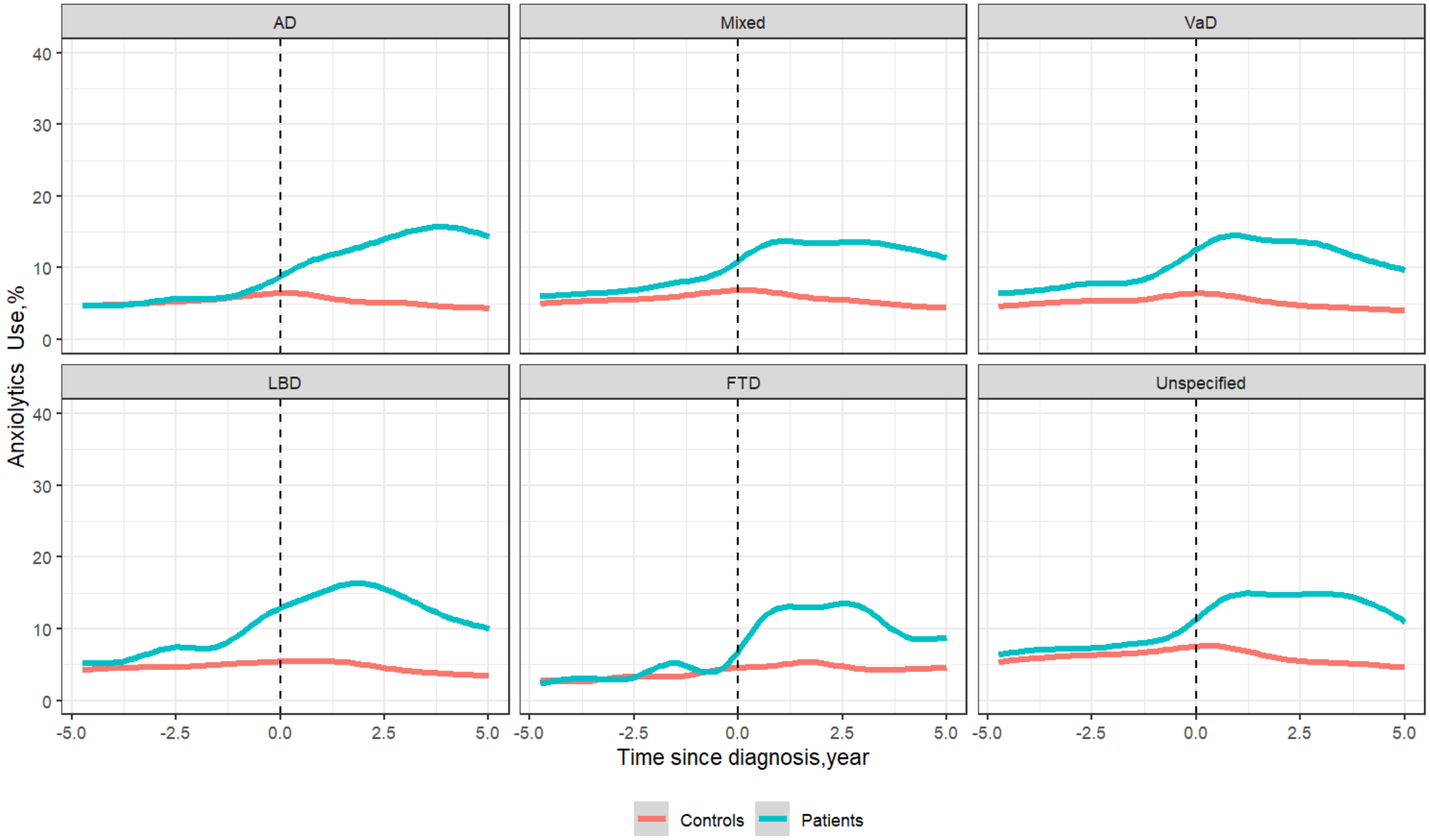

eFigure 7. Use of anxiolytics by dementia types before and after a specific dementia diagnosis in patients from SveDem and matched controls, 2006 to 2017

Abbreviations: AD, Alzheimer's disease; Mixed, mixed dementia; VaD, vascular dementia; LBD, Parkinson's disease with dementia and dementia with Lewy bodies; FTD, frontotemporal dementia; Unspecified, unspecified dementia.

© 2023 Mo M et al. *JAMA Network Open*.

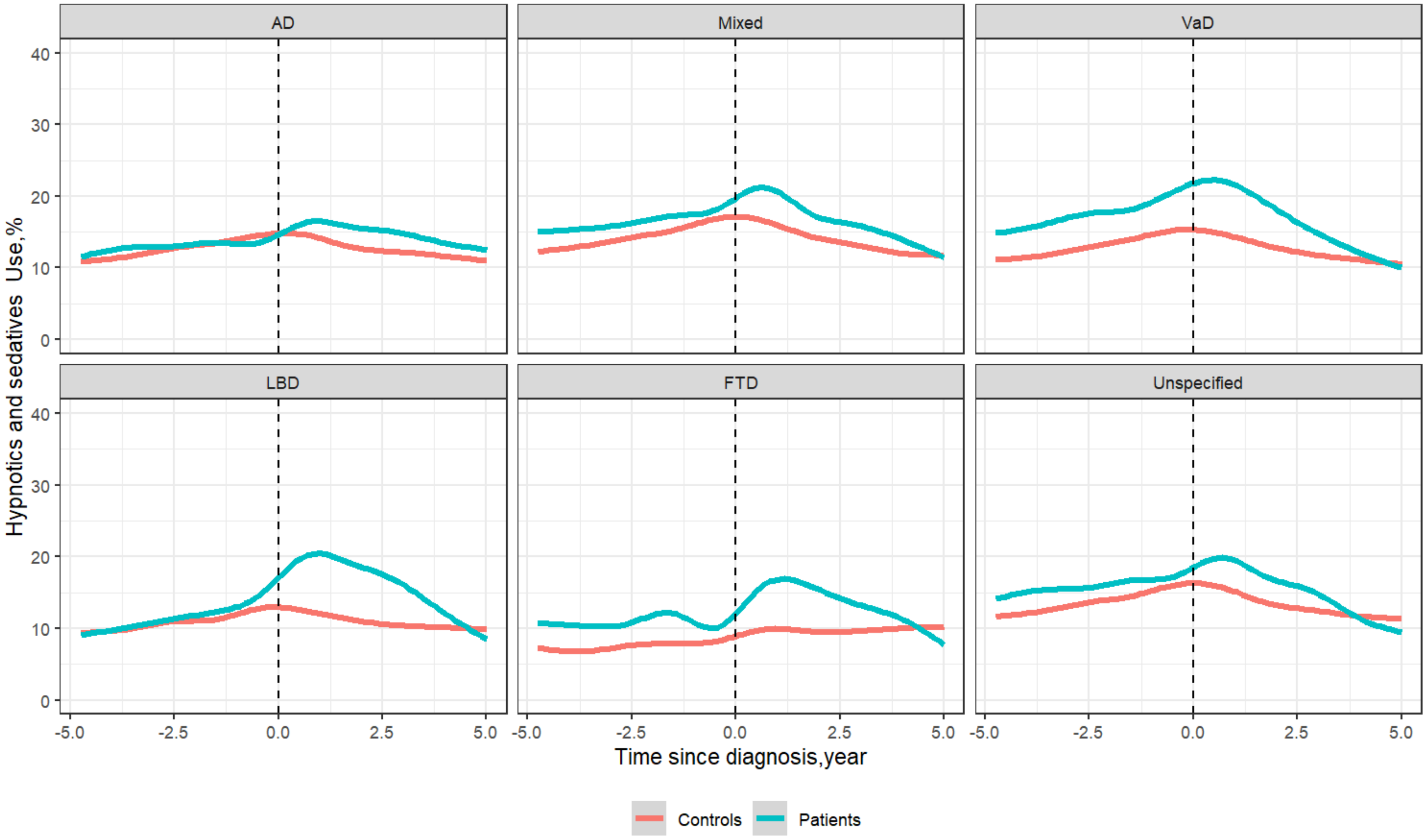

eFigure 8. Use of hypnotics/sedatives by dementia types before and after a specific dementia diagnosis in patients from SveDem and matched controls, 2006 to 2017

Abbreviations: AD, Alzheimer's disease; Mixed, mixed dementia; VaD, vascular dementia; LBD, Parkinson's disease with dementia and dementia with Lewy bodies; FTD, frontotemporal dementia; Unspecified, unspecified dementia.

© 2023 Mo M et al. *JAMA Network Open*.

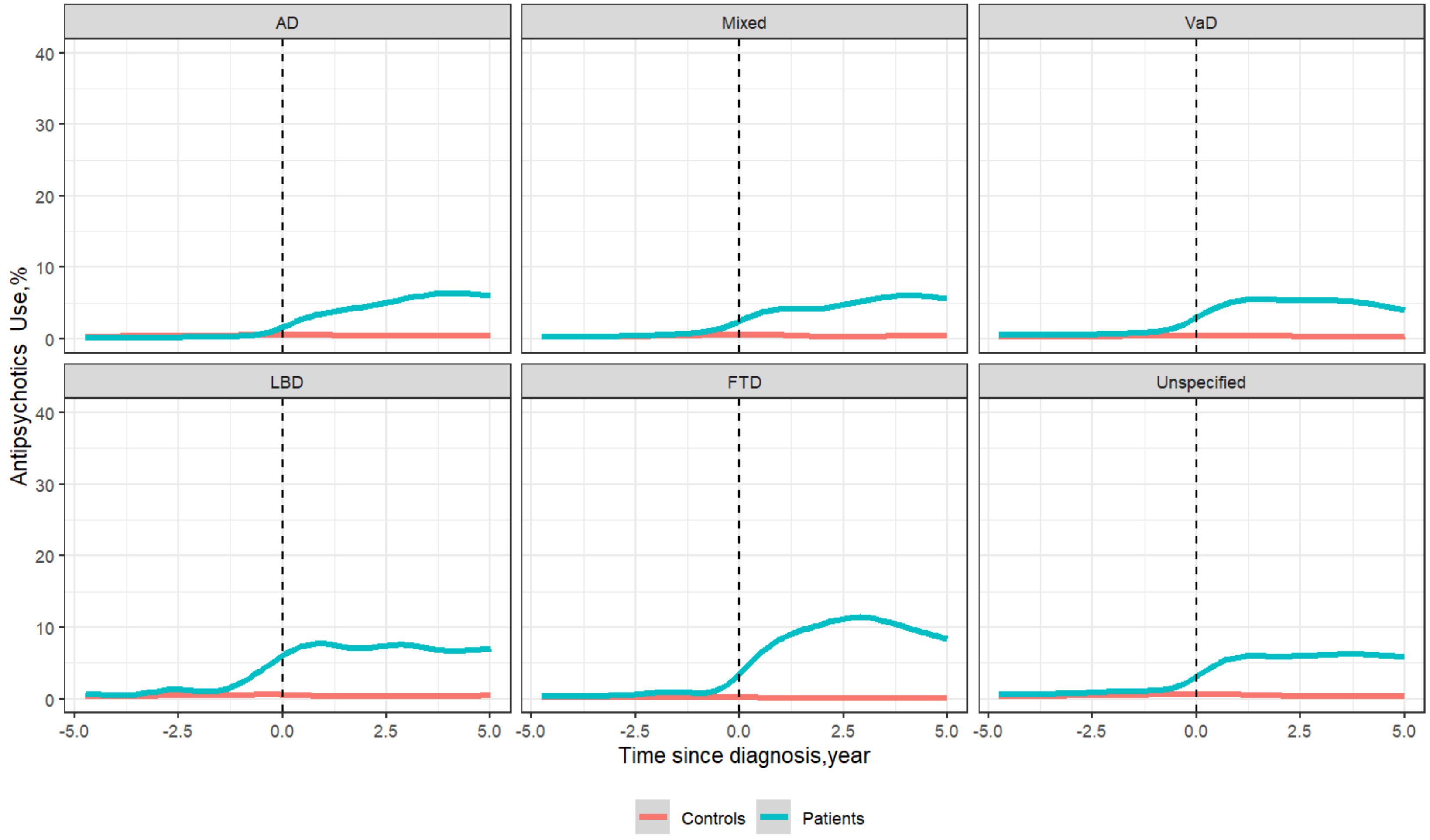

eFigure 9. Use of antipsychotics by dementia types before and after a specific dementia diagnosis in patients from SveDem and matched controls, 2006 to 2017

Abbreviations: AD, Alzheimer's disease; Mixed, mixed dementia; VaD, vascular dementia; LBD, Parkinson's disease with dementia and dementia with Lewy bodies; FTD, frontotemporal dementia; Unspecified, unspecified dementia.

© 2023 Mo M et al. *JAMA Network Open*.

eTable 1. ICD-10 and ATC codes of inclusion criteria for patients with dementia from the National Patient Register and the Swedish Prescribed Drug Register, and exclusion criteria for dementia-free subjects

**Inclusion criteria for patients with dementia**	
National Patient Register	ICD-10 code
Alzheimer's disease, vascular dementia, dementia in other diseases classified elsewhere and unspecified dementia	F00-F03
Alzheimer's disease	G30
Other degenerative diseases of nervous system, not elsewhere classified	G31
Swedish Prescribed Drug Register	ATC code
Anti-dementia drugs	N06D

**Exclusion criteria for dementia-free subjects**	
National Patient Register	ICD-10 code
Delirium, personality and behavioral disorders and other mental disorders due to brain disease, damage and dysfunction and to physical disease, and other unspecified organic or symptomatic mental disorder	F05-F09
Other degenerative disorders of nervous system in diseases classified elsewhere	G32

Abbreviations: ICD-10, International Statistical Classification of Diseases and Related Health Problems, tenth revision; ATC, Anatomical Therapeutic Chemical.

eTable 2. ICD-10 codes for psychiatric disorders

Psychiatric disorers	ICD-10 code
Depression	F32, F33, F34.1, F34.8, F34.9, F38, F39
Anxiety	F40, F41
Stress-related disorders	F43
Substance abuse disorders	F10–F19
Somatoform/conversion disorders	F44-F45
Sleep disorders	F51, G47
Psychotic disorders	F20-29

Abbreviations: ICD-10, International Statistical Classification of Diseases and Related Health Problems, tenth revision.

© 2023 Mo M et al. *JAMA Network Open*.

eTable 3. ATC codes for psychiatric medications

Medications	ATC code
Antidepressants	N06A
Antipsychotics	N05A, excluding N05AH02
Anxiolytics	N05B
Hypnotics/sedatives	N05C

Abbreviations: ATC, Anatomical Therapeutic Chemical.

© 2023 Mo M et al. *JAMA Network Open*.

eTable 4. Characteristics of dementia patients and the control participants at the index date

Characteristics	Control participants	Any dementia	AD	Mixed	VaD	LBD	FTD	Unspecified
	N (%)				N (%)			
N	587260	209245	21779	13000	13067	2496	1031	17867
Age at dementia diagnosis, y								
Mean (SD)	79.6 (8.2)	81.9 (8.3)	78.3 (8.2)	81.7 (6.6)	81.3 (7.2)	76.5 (7.2)	71.1 (8.8)	81.4 (7.7)
< 65	30104 (5.1)	8003 (3.8)	1452 (6.7)	140 (1.1)	320 (2.5)	143 (5.7)	249 (24.2)	571 (3.2)
65 - 74	94330 (16.1)	25025 (12.0)	4550 (20.9)	1522 (11.7)	1646 (12.6)	750 (30.1)	386 (37.4)	2196 (12.3)
≥ 75	462826 (78.8)	176217 (84.2)	15777 (72.4)	11338 (87.2)	11101 (85.0)	1603 (64.2)	396 (38.4)	15100 (84.5)
Sex								
Male	262156 (44.6)	85480 (40.9)	7813 (35.9)	5346 (41.1)	6186 (47.3)	1571 (62.9)	475 (46.1)	7069 (39.6)
Female	325104 (55.4)	123765 (59.2)	13966 (64.1)	7654 (58.9)	6881 (52.7)	925 (37.1)	556 (53.9)	10798 (60.4)
Education attainment								
< 9 years	238399 (40.6)	92927 (44.4)	7407 (34.0)	4581 (35.2)	5461 (41.8)	698 (28.0)	224 (21.7)	8236 (46.1)
9-12 years	236604 (40.3)	81541 (39.0)	9601 (44.1)	5679 (43.7)	5415 (41.4)	1138 (45.6)	502 (48.7)	7021 (39.3)
≥ 13 years	100070 (17.0)	29686 (14.2)	4438 (20.4)	2422 (18.6)	1910 (14.6)	629 (25.2)	295 (28.6)	2227 (12.5)
Missing	12187 (2.1)	5091 (2.4)	333 (1.5)	318 (2.5)	281 (2.2)	31 (1.2)	10 (1.0)	383 (2.1)
Marital status								
Married	275934 (47.0)	84535 (40.4)	10861 (49.9)	5797 (44.6)	5815 (44.5)	1523 (61.0)	585 (56.7)	7369 (41.2)
Unmarried	47057 (8.0)	16392 (7.8)	1421 (6.5)	788 (6.1)	802 (6.1)	148 (5.9)	104 (10.1)	1326 (7.4)

© 2023 Mo M et al. *JAMA Network Open*.

Divorced/seperated	78418 (13.4)	28406 (13.6)	3099 (14.2)	1931 (14.9)	2053 (15.7)	368 (14.7)	214 (20.8)	2510 (14.1)
Widow	185326 (31.6)	79548 (38.0)	6365 (29.2)	4467 (34.4)	4383 (33.5)	450 (18.0)	128 (12.4)	6637 (37.2)
Other	525 (0.1)	364 (0.2)	33 (0.2)	17 (0.1)	14 (0.1)	7 (0.3)	-	25 (0.1)
Disposable individual income (in 100SEK) [a]								
< 1300	144009 (24.5)	55166 (26.4)	5124 (23.5)	2624 (20.2)	2984 (22.8)	424 (17.0)	179 (17.4)	4856 (27.2)
1300-1562	141634 (24.1)	57382 (27.4)	4864 (22.3)	2906 (22.4)	3291 (25.2)	465 (18.6)	187 (18.1)	5018 (28.1)
1562-1966	146400 (24.9)	52767 (25.2)	5577 (25.6)	3853 (29.6)	3852 (29.5)	637 (25.5)	250 (24.3)	4686 (26.2)
≥ 1966	155217 (26.4)	43930 (21.0)	6214 (28.5)	3617 (27.8)	2940 (22.5)	970 (38.9)	415 (40.3)	3307 (18.5)
Calendar year of diagnosis								
2007-2009	137732 (23.5)	49443 (23.6)	3495 (16.1)	1865 (14.4)	1630 (12.5)	397 (15.9)	146 (14.2)	2161 (12.1)
2010-2012	160616 (27.4)	60143 (28.7)	6430 (29.5)	3735 (28.7)	3870 (29.6)	739 (29.6)	316 (30.7)	6116 (34.2)
2013-2015	168574 (28.7)	60354 (28.8)	7210 (33.1)	4540 (34.9)	4760 (36.4)	832 (33.3)	332 (32.2)	6614 (37.0)
2016-2017	120338 (20.5)	39305 (18.8)	4644 (21.3)	2860 (22.0)	2807 (21.5)	528 (21.2)	237 (23.0)	2976 (16.7)
Coresident status								
Cohabiting	276017 (47.0)	84548 (40.4)	10864 (49.9)	5797 (44.6)	5815 (44.5)	1523 (61.0)	585 (56.7)	7369 (41.2)
Living alone	311243 (53.0)	124697 (59.6)	10915 (50.1)	7203 (55.4)	7252 (55.5)	973 (39.0)	446 (43.3)	10498 (58.8)
Region of birth								
Sweden	521521 (88.8)	185271 (88.5)	19195 (88.1)	11151 (85.8)	11298 (86.5)	2247 (90.0)	918 (89.0)	15688 (87.8)
Other	65739 (11.2)	23974 (11.5)	2584 (11.9)	1849 (14.2)	1769 (13.5)	249 (10.0)	113 (11.0)	2179 (12.2)
Charlson Comorbidity Index [b]								
Mean (SD)	2.5 (1.9)	2.3 (1.7)	2.0 (1.4)	2.2 (1.6)	2.3 (1.7)	2.1 (1.5)	1.9 (1.3)	2.1 (1.5)

© 2023 Mo M et al. *JAMA Network Open*.

0	369238 (62.9)	107564 (51.4)	15656 (71.9)	7053 (54.3)	5386 (41.2)	1657 (66.4)	760 (73.7)	10854 (60.8)
1	81535 (13.9)	42578 (20.4)	2901 (13.3)	2628 (20.2)	3084 (23.6)	366 (14.7)	134 (13.0)	3147 (17.6)
2	67146 (11.4)	27156 (13.0)	1945 (8.9)	1644 (12.7)	1956 (15.0)	278 (11.1)	86 (8.3)	1999 (11.2)
≥ 3	69341 (11.8)	31947 (15.3)	1277 (5.9)	1675 (12.9)	2641 (20.2)	195 (7.8)	51 (5.0)	1867 (10.5)
MMSE score [c]								
0-9		1730 (2.5)	504 (2.3)	328 (2.5)	318 (2.4)	53 (2.1)	23 (2.2)	504 (2.8)
10-19		20733 (29.9)	6097 (28.0)	4179 (32.2)	3847 (29.4)	681 (27.3)	187 (18.1)	5742 (32.1)
20-24		25933 (37.5)	8428 (38.7)	5013 (38.6)	4937 (37.8)	958 (38.4)	309 (30.0)	6288 (35.2)
25-30		16304 (23.6)	5904 (27.1)	2953 (22.7)	2986 (22.9)	631 (25.3)	424 (41.1)	3406 (19.1)
Missing		4540 (6.6)	846 (3.9)	527 (4.1)	979 (7.5)	173 (6.9)	88 (8.5)	1927 (10.8)
Type of diagnostic unit [c]								
Specialist care		38290 (55.3)	14321 (65.8)	9932 (76.4)	6620 (50.7)	2192 (87.8)	926 (89.8)	4299 (24.1)
Primary care		30849 (44.6)	7439 (34.2)	3057 (23.5)	6431 (49.2)	302 (12.1)	105 (10.2)	13515 (75.6)
Other unit		101 (0.2)	19 (0.1)	11 (0.1)	16 (0.1)	2 (0.1)	-	53 (0.3)

Abbreviations: AD, Alzheimer's disease; Mixed, mixed dementia; VaD, vascular dementia; LBD, Parkinson's disease with dementia and dementia with Lewy bodies; FTD, frontotemporal dementia; Unspecified, unspecified dementia; SD, standard deviation; MMSE, Mini-Mental State Examination. [a], Disposable income of persons with dementia 1 year before dementia diagnosis was inflated into 2021 values (from Sweden Statistics: Consumer Price Index) with inflation rate from the Swedish Consumer Price Index. The inflated income was then classified into quartiles. [b], Disease status within 3 years before the diagnosis of dementia disease. [c], For patients with dementia from SveDem.

© 2023 Mo M et al. *JAMA Network Open*.

eTable 5. Distribution of psychiatric disorders, person-years and incidence rates a matched cohort study in Sweden, 2000 to 2017

Psychiatric disorders	Control participants			Any dementia		
	Person-years	N	Incidence rate per 1 000 person-years	Person-years	N	Incidence rate per 1 000 person-years
Overall	5947185.5	56634	9.5	1877431.2	37116	19.8
Depression	6124809.8	20532	3.4	1965602.5	18821	9.6
Anxiety	6159886.5	13792	2.2	2012844.9	9197	4.6
Stress-related disorders	6201173.8	3028	0.5	2043301.0	1774	0.9
Substance abuse	6161804.5	12525	2.0	2013514.1	8083	4.0
Somatoform/conversion disorders	6202004.4	2694	0.4	2047380.5	972	0.5
Sleep disorders	6145070.3	13612	2.2	2029686.6	4758	2.3
Psychotic disorders	6204756.5	2389	0.4	2037268.9	3605	1.8

© 2023 Mo M et al. *JAMA Network Open*.

eTable 6. Distribution of psychiatric disorders, person-years and incidence rates in patients from SveDem and matched controls, 2000 to 2017

Psychiatric disorders	Control participants			Any dementia		
	Person-years	N	Incidence rate per 1 000 person-years	Person-years	N	Incidence rate per 1 000 person-years
Overall	2118713.5	20769	9.8	657637.7	12487	19.0
Depression	2186246.2	7079	3.2	688895.7	6301	9.1
Anxiety	2197728.9	4981	2.3	705533.3	3043	4.3
Stress-related disorders	2212365.6	1138	0.5	716129.5	595	0.8
Substance abuse	2197373.9	4799	2.2	706046.0	2679	3.8
Somatoform/conversion disorders	2212775.8	1064	0.5	717344.5	353	0.5
Sleep disorders	2190208.4	5294	2.4	710573.7	1747	2.5
Psychotic disorders	2214221.2	795	0.4	715409.3	897	1.3

© 2023 Mo M et al. *JAMA Network Open*.

eTable 7. Distribution of psychiatric disorders, person-years and incidence rates in patients from other registers and matched controls, 2000 to 2017

Psychiatric disorders	Control participants			Any dementia		
	Person-years	N	Incidence rate per 1 000 person-years	Person-years	N	Incidence rate per 1 000 person-years
Overall	3828472.0	35865	9.4	1219793.6	24629	20.2
Depression	3938563.6	13453	3.4	1276706.8	12520	9.8
Anxiety	3962157.6	8811	2.2	1307311.6	6154	4.7
Stress-related disorders	3988808.2	1890	0.5	1327171.5	1179	0.9
Substance abuse	3964430.6	7726	1.9	1307468.2	5404	4.1
Somatoform/conversion disorders	3989228.5	1630	0.4	1330036.0	619	0.5
Sleep disorders	3954861.9	8318	2.1	1319112.9	3011	2.3
Psychotic disorders	3990535.3	1594	0.4	1321859.6	2708	2.0

© 2023 Mo M et al. *JAMA Network Open*.

eTable 8. Distribution of psychiatric disorders, person-years and incidence rates by a specific dementia diagnosis in patients from SveDem and matched controls, 2000 to 2017

Psychiatric disorders	Control participants			Any dementia		
	Person-years	N	Incidence rate per 1 000 person-years	Person-years	N	Incidence rate per 1 000 person-years
**AD**						
Overall	708351.7	6950	9.8	215400.2	3504	16.3
Depression	731796.2	2285	3.1	223698.2	1884	8.4
Anxiety	735698.6	1616	2.2	229022.6	899	3.9
Stress-related disorders	740233.1	405	0.5	232161.0	177	0.8
Substance abuse	735168.7	1677	2.3	230698.0	493	2.1
Somatoform/conversion disorders	740555.4	386	0.5	232503.1	117	0.5
Sleep disorders	732812.4	1828	2.5	230548.2	500	2.2
Psychotic disorders	741080.7	280	0.4	232109.2	232	1.0
**Mixed**						
Overall	373001.4	3751	10.1	121411.2	2299	18.9
Depression	384522.3	1328	3.5	126776.2	1166	9.2
Anxiety	386519.0	925	2.4	129497.0	602	4.6
Stress-related disorders	389170.5	187	0.5	131596.4	89	0.7
Substance abuse	386624.0	811	2.1	130035.6	446	3.4
Somatoform/conversion disorders	389055.8	192	0.5	131765.5	61	0.5
Sleep disorders	385315.4	898	2.3	130591.6	306	2.3
Psychotic disorders	389325.2	142	0.4	131372.6	162	1.2
**VaD**						

© 2023 Mo M et al. *JAMA Network Open*.

Overall	382896.5	3824	10.0	118604.8	2667	22.5
Depression	395067.2	1296	3.3	125155.4	1317	10.5
Anxiety	396885.6	930	2.3	128614.4	623	4.8
Stress-related disorders	399622.1	208	0.5	130616.4	137	1.0
Substance abuse	397039.6	846	2.1	128204.6	658	5.1
Somatoform/conversion disorders	399643.4	191	0.5	130934.2	59	0.5
Sleep disorders	395297.1	997	2.5	129352.5	403	3.1
Psychotic disorders	399988.5	141	0.4	130678.9	146	1.1
**LBD**						
Overall	87919.9	900	10.2	22533.7	604	26.8
Depression	91337.2	250	2.7	23873.6	324	13.6
Anxiety	91778.7	175	1.9	24695.6	146	5.9
Stress-related disorders	92303.5	41	0.4	25273.5	24	0.9
Substance abuse	91321.8	263	2.9	25162.0	48	1.9
Somatoform/conversion disorders	92345.1	36	0.4	25272.6	26	1.0
Sleep disorders	90951.4	291	3.2	24860.5	120	4.8
Psychotic disorders	92393.6	23	0.2	25176.4	64	2.5
**FTD**						
Overall	38297.9	424	11.1	9377.9	269	28.7
Depression	40022.7	136	3.4	10036.8	138	13.7
Anxiety	40223.4	100	2.5	10461.7	58	5.5
Stress-related disorders	40522.4	39	1.0	10650.7	23	2.2
Substance abuse	40176.2	114	2.8	10409.1	69	6.6
Somatoform/conversion disorders	40647.2	19	0.5	10721.0	7	0.7
Sleep disorders	39989.2	131	3.3	10538.1	48	4.6
Psychotic disorders	40604.2	19	0.5	10673.4	27	2.5
**Unspecified**						
Overall	528246.2	4920	9.3	170309.8	3144	18.5

Depression	543500.6	1784	3.3	179355.6	1472	8.2
Anxiety	546623.6	1235	2.3	183242.0	715	3.9
Stress-related disorders	550514.0	258	0.5	185831.5	145	0.8
Substance abuse	547043.7	1088	2.0	181536.7	965	5.3
Somatoform/conversion disorders	550528.9	240	0.4	186148.0	83	0.4
Sleep disorders	545842.9	1149	2.1	184682.7	370	2.0
Psychotic disorders	550829.0	190	0.3	185398.8	266	1.4

Abbreviations: AD, Alzheimer's disease; Mixed, mixed dementia; VaD, vascular dementia; LBD, Parkinson's disease with dementia and dementia with Lewy bodies; FTD, frontotemporal dementia; Unspecified, unspecified dementia.

© 2023 Mo M et al. *JAMA Network Open*.

eTable 9. Hazard ratios and 95% CIs of psychiatric disorders before a specific dementia diagnosis in patients form SveDem and matched controls, 2000 to 2017[a]

	Risk before diagnosis, HR (95% CI)					
Dementia categories	-6.5 y	-5 y	-3 y	-2 y	-1 y	-0.5 y
AD	1.23 (1.06-1.44)	1.15 (1.05-1.25)	1.36 (1.24-1.48)	2.10 (1.97-2.25)	2.95 (2.73-3.19)	3.16 (2.89-3.45)
Mixed	1.54 (1.28-1.85)	1.34 (1.20-1.48)	1.42 (1.27-1.59)	2.06 (1.90-2.24)	2.85 (2.58-3.15)	3.00 (2.68-3.36)
VaD	1.37 (1.15-1.62)	1.42 (1.29-1.57)	1.72 (1.56-1.91)	2.32 (2.15-2.51)	2.88 (2.61-3.17)	2.98 (2.67-3.32)
LBD	1.26 (0.86-1.85)	1.66 (1.33-2.08)	2.14 (1.69-2.70)	3.72 (3.11-4.45)	5.77 (4.72-7.05)	5.95 (4.75-7.45)
FTD	1.26 (0.69-2.28)	1.21 (0.88-1.66)	1.45 (1.04-2.01)	2.78 (2.17-3.56)	6.07 (4.54-8.12)	7.08 (4.99-10.05)
Unspecified	1.88 (1.62-2.19)	1.54 (1.41-1.68)	1.72 (1.57-1.88)	2.64 (2.45-2.83)	3.62 (3.31-3.95)	3.86 (3.49-4.26)

Abbreviations: AD, Alzheimer's disease; Mixed, mixed dementia; VaD, vascular dementia; LBD, Parkinson's disease with dementia and dementia with Lewy bodies; FTD, frontotemporal dementia; Unspecified, unspecified dementia.

[a] All models were adjusted for age, sex, educational attainment (< 9 years, 9-12 years, ≥ 13 years, Missing), disposable individual income (in 100 SEK) (<1300, 1300-1562, 1562-1966, ≥1966), region of birth (Sweden or other), Charlson Comorbidity Index (0, 1, 2, ≥3), and calendar year of diagnosis (2007-2009, 2010-2012, 2013-2015, or 2016-2017).

© 2023 Mo M et al. *JAMA Network Open*.

eTable 10. Hazard ratios and 95% CIs of psychiatric disorders after a specific dementia diagnosis in patients from SveDem and matched controls, 2000 to 2017[a]

Dementia categories	Risk after diagnosis, HR (95% CI)								
	1 wk	0.5 y	1 y	2 y	3 y	5 y	6.5 y	8 y	10 y
AD	2.88 (1.92-4.34)	2.12 (1.88-2.39)	1.74 (1.58-1.91)	1.32 (1.18-1.49)	1.13 (1.03-1.25)	0.85 (0.73-1.00)	0.74 (0.59-0.91)	0.66 (0.51-0.86)	0.60 (0.45-0.81)
Mixed	3.15 (1.72-5.76)	2.17 (1.85-2.55)	1.76 (1.54-2.00)	1.33 (1.14-1.55)	1.10 (0.96-1.27)	0.83 (0.65-1.05)	0.72 (0.52-0.98)	0.65 (0.45-0.94)	0.59 (0.39-0.89)
VaD	3.35 (2.15-5.21)	2.32 (1.99-2.70)	1.92 (1.68-2.20)	1.56 (1.35-1.81)	1.35 (1.18-1.55)	1.14 (0.92-1.42)	1.06 (0.80-1.39)	0.99 (0.72-1.37)	0.93 (0.65-1.33)
LBD	6.69 (3.11-14.41)	3.20 (2.29-4.47)	2.32 (1.78-3.04)	1.66 (1.19-2.31)	1.54 (1.16-2.05)	1.59 (1.02-2.47)	1.60 (0.89-2.90)	1.60 (0.80-3.22)	1.58 (0.71-3.48)
FTD	6.12 (1.49-25.14)	3.15 (1.80-5.53)	2.00 (1.27-3.14)	1.29 (0.71-2.34)	1.31 (0.81-2.11)	1.29 (0.65-2.57)	1.25 (0.51-3.06)	1.21 (0.44-3.32)	1.16 (0.39-3.49)
Unspecified	5.23 (3.43-7.99)	2.02 (1.75-2.32)	1.67 (1.48-1.88)	1.33 (1.16-1.53)	1.13 (1.00-1.27)	0.94 (0.77-1.13)	0.85 (0.67-1.09)	0.79 (0.60-1.05)	0.73 (0.53-1.01)

Abbreviations: AD, Alzheimer's disease; Mixed, mixed dementia; VaD, vascular dementia; LBD, Parkinson's disease with dementia and dementia with Lewy bodies; FTD, frontotemporal dementia; Unspecified, unspecified dementia.

[a], All models were adjusted for age, sex, educational attainment (< 9 years, 9-12 years, ≥ 13 years, Missing), disposable individual income (in 100 SEK) (<1300, 1300-1562, 1562-1966, ≥1966), region of birth (Sweden or other), Charlson Comorbidity Index (0, 1, 2, ≥3), and calendar year of diagnosis (2007-2009, 2010-2012, 2013-2015, or 2016-2017).

© 2023 Mo M et al. *JAMA Network Open*.

eTable 11. Use of antidepressants, anxiolytics, hypnotics/sedatives and antipsychotics before and after dementia diagnosis in a matched cohort study in Sweden, 2006 to 2017[a]

	Time since diagnosis, year												
Use of psychiatric medications	-4.5 y	-4 y	-3 y	-2 y	-1 y	-0.5 y	0 y	0.5 y	1 y	2 y	3 y	4 y	5 y
Antidepressants use, %													
Control	6.4	6.7	0.7	7.9	8.7	9.2	9.9	9.7	9.0	8.1	7.6	7.2	6.9
Case	10.9	11.9	13.5	15.9	19.4	22.6	27.9	29.1	28.4	26.4	23.7	20.2	16.4
Anxiolytics use, %													
Control	5.3	5.5	5.7	6.0	6.6	6.8	7.3	7.2	6.6	5.8	5.1	5.0	4.3
Case	7.0	7.4	8.0	8.8	10.4	11.6	15.3	15.2	14.1	12.9	11.9	10.3	8.5
Hypnotics/sedatives use, %													
Control	12.2	12.5	13.4	14.5	15.5	16.1	16.6	16.1	15.0	13.3	12.2	11.2	10.5
Case	15.2	15.8	16.4	17.2	17.9	18.5	20.9	20.0	18.0	14.5	11.8	9.5	7.6
Antipsychotics use, %													
Control	0.3	0.3	0.4	0.4	0.5	0.6	0.6	0.6	0.5	0.4	0.4	0.3	0.4
Case	0.9	1.1	1.3	1.6	2.3	3.1	5.0	5.7	5.7	5.3	5.0	4.7	4.1

[a], *P* values for difference among patients with dementia and control participants are all significant at $P < .001$ based on χ^2 tests with Bonferroni correction, except use of hypnotics/sedatives at 3 years after diagnosis.

© 2023 Mo M et al. *JAMA Network Open*.

eReferences

1. Religa D, Fereshtehnejad SM, Cermakova P, et al. SveDem, the Swedish Dementia Registry - a tool for improving the quality of diagnostics, treatment and care of dementia patients in clinical practice. *PLoS One*. 2015;10(2):e0116538. doi:10.1371/journal.pone.0116538

2. Ludvigsson JF, Andersson E, Ekbom A, et al. External review and validation of the Swedish national inpatient register. *BMC Public Health*. Jun 9 2011;11:450. doi:10.1186/1471-2458-11-450

3. Wettermark B, Hammar N, Fored CM, et al. The new Swedish Prescribed Drug Register--opportunities for pharmacoepidemiological research and experience from the first six months. *Pharmacoepidemiol Drug Saf*. Jul 2007;16(7):726-35. doi:10.1002/pds.1294

4. Barnes TR, Schizophrenia Consensus Group of British Association for P. Evidence-based guidelines for the pharmacological treatment of schizophrenia: recommendations from the British Association for Psychopharmacology. *J Psychopharmacol*. May 2011;25(5):567-620. doi:10.1177/0269881110391123

5. Leucht S, Cipriani A, Spineli L, et al. Comparative efficacy and tolerability of 15 antipsychotic drugs in schizophrenia: a multiple-treatments meta-analysis. *Lancet*. Sep 14 2013;382(9896):951-62. doi:10.1016/S0140-6736(13)60733-3

6. Fazel S, Zetterqvist J, Larsson H, Langstrom N, Lichtenstein P. Antipsychotics, mood stabilisers, and risk of violent crime. *Lancet*. Sep 27 2014;384(9949):1206-14. doi:10.1016/S0140-6736(14)60379-2

© 2023 Mo M et al. *JAMA Network Open*.
