# Supplementary material for: Psychiatric Disorders Before and After Dementia Diagnosis
Source: JAMA Netw Open. 2023 Oct 17;6(10):e2338080. doi: 10.1001/jamanetworkopen.2023.38080 (PMC10582787; doi:10.1001/jamanetworkopen.2023.38080)
Supplement: Supplement 2. — Data Sharing Statement [file jamanetwopen-e2338080-s002.pdf]

Data Sharing Statement

Mo. Psychiatric disorders before and after dementia diagnosis. *JAMA Netw Open*. Published October 17, 2023. doi:10.1001/jamanetworkopen.2023.38080

Data
Data available: No

Additional Information
Explanation for why data not available: Data not available due to ethical restrictions.
